# An atlas of human vector-borne microbe interactions reveals pathogenicity mechanisms

**DOI:** 10.1016/j.cell.2024.05.023

**Published:** 2024-06-13

**Authors:** Thomas M. Hart, Nicole D. Sonnert, Xiaotian Tang, Reetika Chaurasia, Paige E. Allen, Jason R. Hunt, Curtis B. Read, Emily E. Johnson, Gunjan Arora, Yile Dai, Yingjun Cui, Yu-Min Chuang, Qian Yu, M. Sayeedur Rahman, M. Tays Mendes, Agustin Rolandelli, Pallavi Singh, Abhai K. Tripathi, Choukri Ben Mamoun, Melissa J. Caimano, Justin D. Radolf, Yi-Pin Lin, Volker Fingerle, Gabriele Margos, Utpal Pal, Raymond M. Johnson, Joao H.F. Pedra, Abdu F. Azad, Jeanne Salje, George Dimopoulos, Joseph M. Vinetz, Jason A. Carlyon, Noah W. Palm, Erol Fikrig, Aaron M. Ring

**Affiliations:** 1 Dept. of Internal Medicine, Yale School of Medicine, New Haven, CT, 06510, USA; 2 Dept. of Immunobiology, Yale School of Medicine, New Haven, CT, 06510, USA; 3 Dept. of Microbial Pathogenesis, Yale School of Medicine, New Haven, CT, 06510, USA; 4 Dept. of Microbiology and Immunology, Virginia Commonwealth University School of Medicine, Richmond, VA, 23298, USA; 5 Dept. of Epidemiology and Microbial Diseases, Yale School of Public Health, New Haven, CT, 06510, USA; 6 Dept. of Microbiology and Immunology, University of Maryland School of Medicine, Baltimore, MD, USA; 7 W. Harry Feinstone Dept. of Molecular Microbiology and Immunology, Bloomberg School of Public Health, Johns Hopkins University, Baltimore, MD, 21205, USA; 8 Dept. of Medicine, UConn Health, Farmington, CT, 06030, USA; 9 Dept. of Pediatrics, UConn Health, Farmington, CT, 06030, USA; 10 Dept. of Molecular Biology and Biophysics, UConn Health, Farmington, CT, 06030, USA; 11 Dept. of Genetics and Genome Sciences, UConn Health, Farmington, CT, 06030, USA; 12 Dept. of Immunology, UConn Health, Farmington, CT, 06030, USA; 13 Division of Infectious Diseases, Wadsworth Center, New York State Dept. of Health, Albany, NY, 12201, USA; 14 Bavarian Health and Food Safety Authority, Oberschleiβheim, Bavaria, 85764, DEU; 15 Dept. of Veterinary Medicine, University of Maryland, College Park, MD, 20742, USA; 16 Dept. of Pathology, University of Cambridge, Cambridge, CB2 1TN, UK; 17 Dept. of Biochemistry, University of Cambridge, Cambridge, CB2 1TN, UK; 18 Laboratorio ICEMR-Amazonia, Laboratorios de Investigación Y Desarrollo, Facultad de Ciencias Y Filosofia, Universidad Peruana Cayetano Heredia, Lima, 15102, Peru; 19 Instituto de Medicina Tropical Alexander Von Humboldt, Universidad Peruana Cayetano Heredia, Lima, Lima, 15102, Peru; 20 Translational Science and Therapeutics Division, Fred Hutchinson Cancer Center, Seattle, Washington, 98102, USA

**Keywords:** Host-pathogen interactions, vector-borne disease, arthropod-borne disease, infectious disease, mechanisms of pathogenicity, protein disulfide isomerase, thioredoxin, systems biology

## Abstract

Vector-borne diseases are a leading cause of death worldwide and pose a substantial unmet medical need. Pathogen binding to host extracellular proteins (the “exoproteome”) represents a crucial interface in the etiology of vector-borne disease. Here, we used BASEHIT—a technique enabling interrogation of microbial interactions with 3,324 human exoproteins—to profile interactomes of 82 human pathogen samples, including 30 strains of arthropod-borne pathogens and eight strains of related non-vector borne pathogens. The resulting atlas revealed 1,303 putative interactions, including hundreds of pairings with potential roles in pathogenesis, including cell invasion, tissue colonization, immune evasion, and host sensing. Subsequent functional investigations uncovered that Lyme disease spirochetes recognize epidermal growth factor as an environmental cue of transcriptional regulation and that conserved interactions between intracellular pathogens and thioredoxins facilitates cell invasion. In summary, this interactome atlas provides molecular-level insights into microbial pathogenesis and reveals potential host directed targets for next-generation therapeutics.

## IN BRIEF

A curated yeast display library expressing 3,324 human extracellular and secreted proteins was probed with 82 diverse pathogen samples, resulting in a foundational database of 1,303 putative interactions that sheds light on potential mechanisms of pathogenesis.

## Graphical Abstract

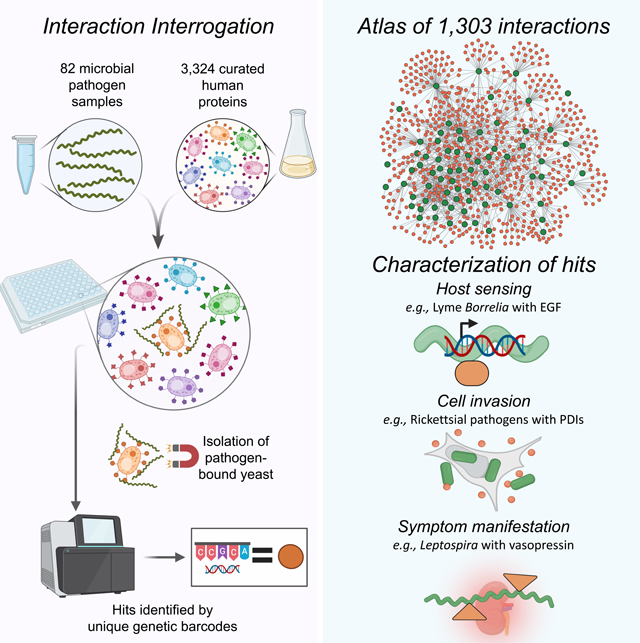

## INTRODUCTION

At least two-thirds of the world’s population is currently at risk from vector-borne diseases that account for more than one million deaths annually. Since 2014, major outbreaks of dengue, malaria, chikungunya, yellow fever, and Zika have claimed millions of lives and overwhelmed health systems in many countries. The burden of vector-borne disease will likely increase, as global warming, mass urbanization and transport alter the worldwide distribution of vectors, including mosquitoes, ticks, and sandflies, and associated diseases. A deeper understanding of the pathogenesis of vector-borne diseases is thus urgently needed to develop vaccines and therapeutics against vector-borne pathogens.

Direct interactions between arthropod-borne microbial pathogens and human extracellular and secreted proteins (exoproteins) are crucial and early interfaces shaping vector-borne disease, and the evolution of these interactions has led to the development of varied pathogeneses. For example, many microbes directly bind host extracellular matrix (ECM) proteoglycans or cell surface receptors to facilitate tissue colonization ^1–4^ or cell invasion ^3,4^. Human exoproteins also play crucial roles in coordinating the immune response to invading pathogens. Soluble proteins, such as complement and antimicrobial peptides, bind microbes to opsonize or directly kill them ^5–7^, while receptors on the surface of immune cells, such as toll-like receptors and C-type lectin receptors (TLRs, CLRs), interact with pathogen components to stimulate the immune system ^8,9^. Microbes have also evolved mechanisms to evade recognition by these proteins, which is necessary for their pathogenic potential ^5,6^. For example, many pathogens have evolved diverse cellular components that do not stimulate immune cell receptors, including varied membrane structures ^10^, and mimics of host molecules ^11,12^. Due to the importance of these interactions in the development of disease, detailed characterization of the myriad physical interaction nodes between pathogens and host extracellular proteins is crucial for a mechanistic understanding of microbial pathogenesis. Further, such characterizations would highlight valuable therapeutic targets to treat and prevent infectious disease.

**Figure 1.**
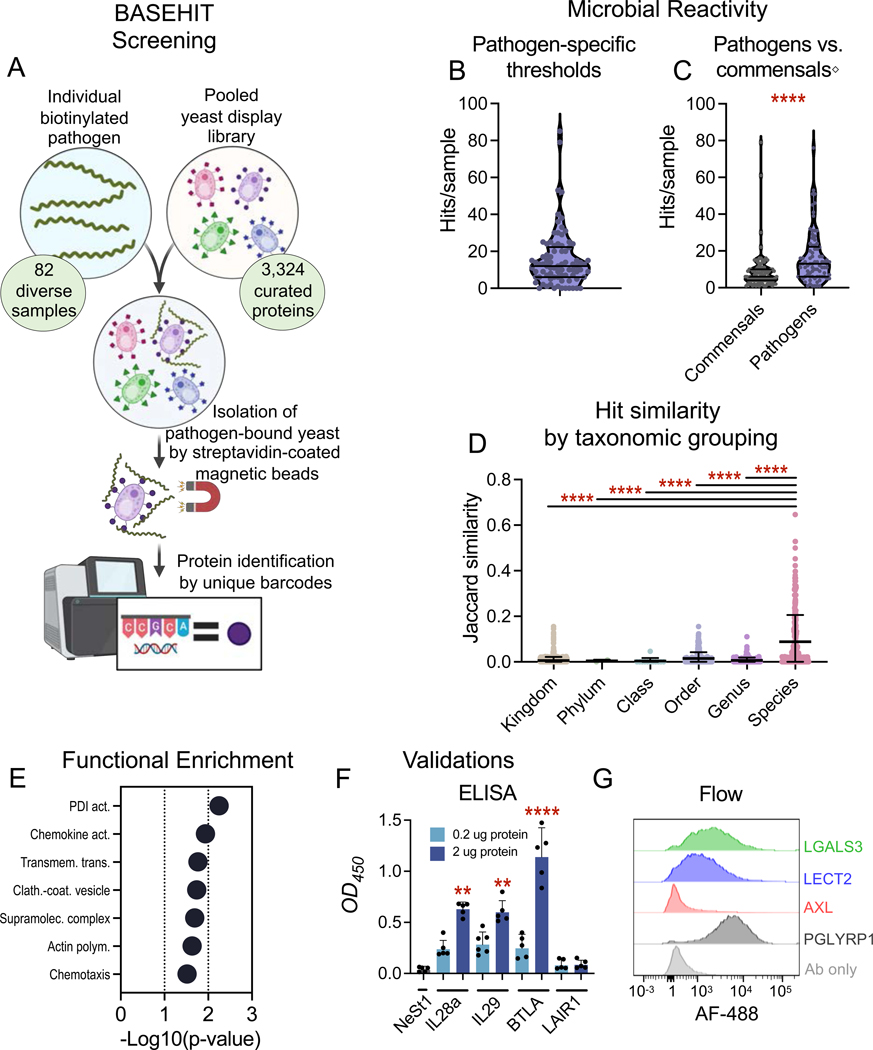
Atlas of pathogen: host exoprotein interactions. **(A)** A diagram of the BASEHIT method illustrates how individual biotinylated pathogen samples were applied to a yeast display library consisting of 3,324 unique human extracellular proteins or protein epitopes. Pathogen-yeast complexes were selected by magnetic isolation using streptavidin-coated magnetic beads. Pathogen-enriched clones were identified by next generation sequencing of clone-specific barcode sequences. **(B, C)** The number of hits for each pathogen and commensal sample are shown with a violin plot showing the mean and quartiles. Hits are defined as human proteins with normalized enrichment scores above **(B)** a pathogen-specific threshold or **(C)** a global threshold of 5 in at least 2 of 3 replicates. Pathogens bound to significantly more human exoproteins than commensals (Mann-Whitney test, p-value < 0.0001). ^◇^Commensal screens detailed in ^18^. **(D)** Samples of the same species tend to have more similar sets of human protein interacting partners, with significantly higher Jaccard similarity than other groupings (Kruskal-Wallis test with a Dunn’s multiple comparisons; p-values < 0.0001). There were no pairings of samples within the same family that were not of the same genus or species **(E)** Clusters of Gene Ontology (GO) biological processes (BP) and molecular functions (MF) enriched among the pathogen-binding human proteins are shown. Significantly enriched GO Terms were clustered based on similarity of protein makeup (κ > 0.5) and functional annotation. Clusters are plotted to the average -Log_10_(p-value) of each included GO Term. **(F)** ELISA validations of *Borrelia* binding proteins. *B. burgdorferi* B31 lysate was coated on microtiter wells in and probed with 0.2 (light blue) or 2 μg (dark blue) 6xHis-tagged IL28a, IL29, B- and T-Lymphocyte Attenuator (BTLA), Leukocyte Associated Immunoglobulin Like Receptor 1 (LAIR1), or the irrelevant 6xHis-tagged protein NeSt1 (gray), and stained with anti-His-HRP antibodies. Binding was determined by colorimetric analysis with TMB substrate. Values represent the geometric mean ± geometric standard deviation of three replicates, and significance was determined by a Kruskal-Wallis test with a Dunn’s multiple comparisons test (** = p-value < 0.005, **** = p-value < 0.0001). **(G)** Flow cytometric validations of *B. burgdorferi* N40 binding proteins were conducted by incubating *B. burgdorferi* N40 grown to 10^7^ spirochetes/ml with 2 μg 6xHis-tagged LGALS3, LECT2, and AXL, as well as the positive control PGLYRP1 or negative control no protein (Ab alone) followed by staining with anti-His-AF-488 antibodies. Data shown are representative of at least 2 independent experiments.

High throughput protein screening systems – such as protein microarrays, phage display, and yeast surface display – are all widely used to produce and probe large sets of proteins ^13^. As eukaryotes, yeast in particular have the advantage of inherently producing the post translational modification machinery required to efficiently secrete properly folded and glycosylated eukaryotic proteins ^14,15^. To enable the comprehensive screening of host-microbe interactions, we developed a technology termed BASEHIT (BActerial Selection to Elucidate Host-microbe Interactions in high Throughput; ^16–18^). This technology leverages a genetically barcoded yeast display library containing 3,324 curated human exoproteins to identify human-microbe interactions (Fig. 1A; Table S1) ^19^. Proteins of interest are displayed on the surface of yeast as a fusion to an endogenous yeast surface protein, Aga2 [38], and the pooled library is probed with individual pathogen samples. Bound protein identities and degrees of enrichment are determined by next-generation sequencing of protein-specific barcodes, enabling multiplexing of hundreds of samples simultaneously for high-throughput screening [38]. Further, the use of a curated and normalized library ensures that the exoproteome is comprehensively sampled. Control screens have demonstrated that BASEHIT has comparable sensitivity to ELISA and exhibits limited off-target binding ^16,19^.

We previously conducted targeted BASEHIT profiling of *Borrelia*, revealing Lyme *Borrelia* bind to a peptidoglycan recognition protein (PGLYRP1) ^16^ and that relapsing fever *Borrelia* bind to the complement inhibitor CD55 ^17^. Furthermore, mice deficient in PGLYRP1 or CD55 exhibited higher Lyme or lower relapsing fever *Borrelia* burdens, respectively, than wildtype mice, demonstrating that BASEHIT can uncover host-microbe interactions that directly influence bacterial pathogenesis. These earlier studies used a preliminary library containing 1,031 exoproteins that only partially sampled the exoproteome. Moreover, PGLYRP1 and CD55 were the only *Borrelia*-binding proteins identified in these screens. These spirochetes are known to bind to many additional exoproteins ^20–23^, indicating that our initial screening and analysis methods (optimized for commensal bacteria ^18^) could benefit from improvements in sensitivity to detect host-pathogen interactions.

Here, we screened the expanded BASEHIT library with diverse human microbial pathogen samples, with a focus on arthropod-borne pathogens. These screen results and subsequent functional investigations reveal interactions that underpin mechanisms of pathogenesis for vector-borne diseases and suggest potential next-generation therapeutic targets to treat a wide range of human infectious diseases.

## RESULTS

### Identification of human exoprotein interactions with a diverse collection of pathogens

Using BASEHIT, we assessed host-microbe interactomes of 82 unique samples of human pathogens. We focused on common microbial vector-borne pathogens, as well as a selection of related, but not vector-borne, microbes. For example, we included the spirochetes that cause the vector-borne illness, Lyme disease, and spirochetes that cause Leptospirosis. We included many vector-borne Rickettsial intracellular pathogens, as well as other non-vector-borne intracellular bacteria like *Chlamydia*. These screens encompassed eukaryotic and prokaryotic pathogens, such as *Plasmodium* and *Rickettsia*, and intracellular and extracellular specialists, such as *Anaplasma* and Lyme *Borrelia*. We grew many of these pathogens under multiple culture conditions known to modify microbial surface protein expression. For example, *Leptospira* was exposed to different osmolarities and temperatures (37°C supplemented with 120 mM NaCl or 30°C without NaCl), Lyme *Borrelia* was exposed to temperatures found in the host (37°C), tick (23°C), or intermediary temperatures (26°C, 30°C, and 33°C), and *Orientia tsutsugamushi* was isolated before and after cell invasion (Detailed in Table 1).

To identify putative pathogen interacting partners, we developed a pathogen-tailored enrichment analysis method (Fig. S1) and set specific score thresholds for each individual pathogen using the scores of known binding partners detected in the dataset (Fig. S2). Using this method, we identified 1,303 putative pathogen-exoprotein interactions encompassing 713 unique human proteins (Detailed in Table S2), for an average of 16 interactions per sample (Fig. 1B). Further, we applied this method to our prior screens of commensal microbes ^18^ and found that pathogens bound nearly twice as many exoproteins on average as compared to commensals (Fig. 1C; p-value < 0.0001). This finding highlights the distinct human-pathogen and human-commensal interaction profiles arising from their divergent evolutionary relationships. The identified exoprotein-pathogen interactions were largely sample-specific, with 488 proteins binding to just one pathogen sample. This result is consistent with expected trends, as each sample represented a distinct combination of pathogen strain and growth condition or life stage. When we removed these sample-specific interacting proteins, we found that pathogen samples of the same species bound to similar sets of exoproteins, as indicated by significantly higher Jaccard similarities than pairings of broader taxonomic groupings (Fig. 1D, see Fig. S3A for dendrogram; p-values < 0.0001). This supports our expectation that pathogens of the same species would have conserved features enabling the binding of similar host proteins. Gene ontology (GO) Term analyses of the molecular functions and biological pathways significantly enriched by the identified pathogen-interacting human proteins demonstrated a wide range of roles played by these bound exoproteins (Fig. 1E, S3B) including protein disulfide isomerase (PDI) activity, innate and adaptive immune responses, and lipid or protein binding. The observed enrichment patterns indicate that these screens have uncovered interactions spanning a broad spectrum of functions.

### Validations of putative interactions

**Figure 2.**
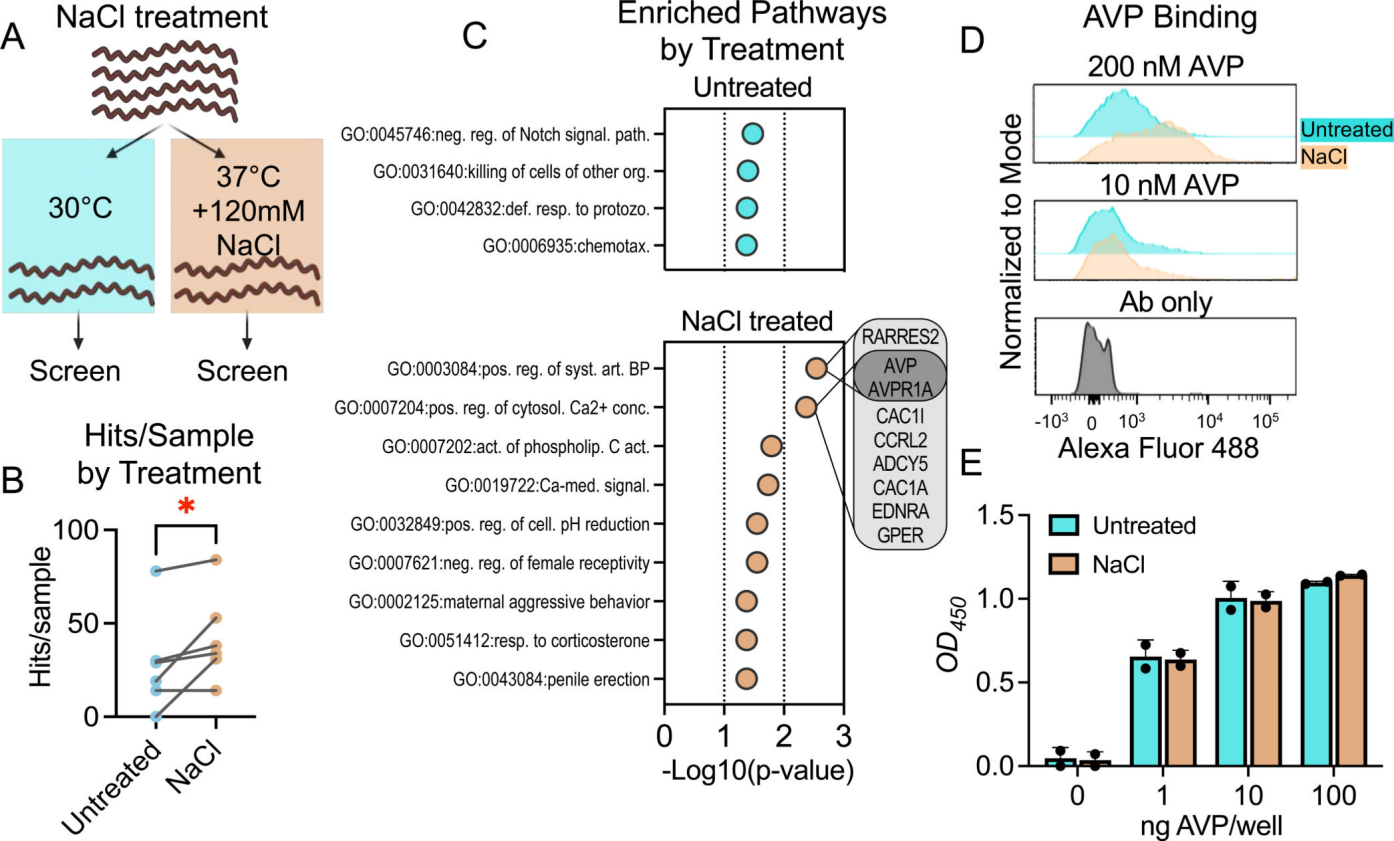
Analysis of exoprotein hits and enriched GO biological pathways by *Leptospira*. **(A)** *Leptospira interrogans* and *biflexa* were grown at 30°C, then maintained at 30°C (traditional culture conditions) or exposed to 37°C + 120 mM NaCl (to simulate host environment temperature and osmolality) for 4 hours. **(B)** The number of hits per sample for untreated or NaCl-treated *L. interrogans* and *biflexa* strains are shown, and samples of the same strain are paired by lines. As expected, host-adapted *Leptospira* bound to significantly more samples than untreated (*, one-tailed Wilcoxon test p-value = 0.03). **(C)** GO BPs significantly enriched by untreated or NaCl-treated *Leptospira* were determined by DAVID and plotted against -Log_10_(p-value) (EASE; p-value < 0.05). A Venn diagram listing the *Leptospira*-binding proteins in the top two pathways enriched by NaCl-binding Leptospira is shown to the right. **(D)** Flow cytometric and **(E)** ELISA validations of *Leptospira*-vasopressin (“AVP”) binding. **(D)** 10^7^ spirochetes were incubated with 0 (“Ab only”), 10 or 200 nM of vasopressin (AVP), followed by an anti-vasopressin antibody and a PE-conjugated secondary antibody. **(E)** ELISA plates (n=2) were coated with 1, 10, or 100 ng vasopressin, or with 100 ng BSA per well, followed by 10^8^ biotinylated host-adapted (tan) or untreated (blue) *L. interrogans* serovar Copenhageni L1–130. Binding was determined by HRP-conjugated streptavidin, and the lowest background reactivity between the *Leptospira* and BSA was subtracted from all samples.

**Figure 3.**
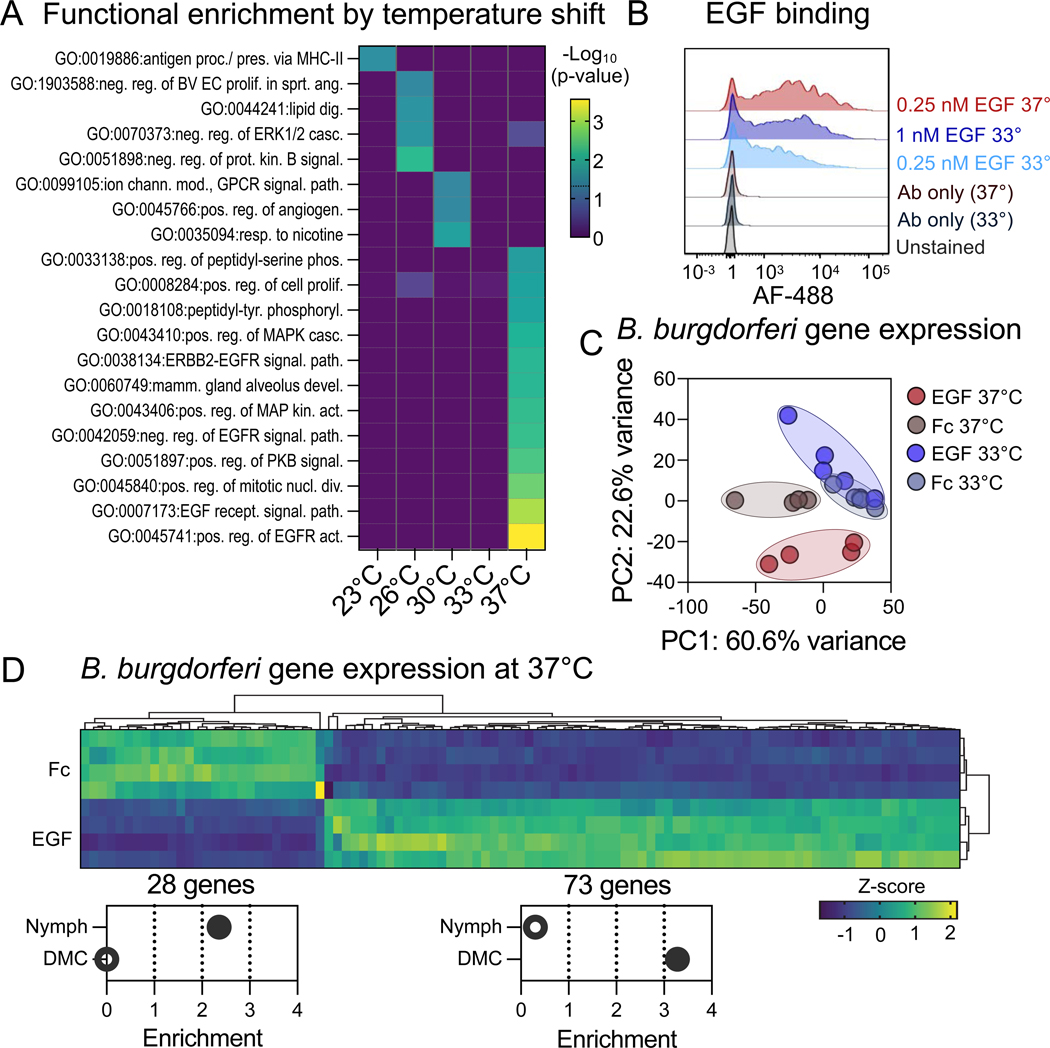
Impact of EGF on *B. burgdorferi* gene expression. **(A)** GO BPs significantly enriched by the temperature shifted *Borrelia* were determined by EASE with DAVID, and are plotted against the temperature treatment. Colors correspond with the --Log_10_(p-value) per the legend to the right, with significance indicated by the dotted line. **(B)** Flow cytometric validations were conducted by incubating *B. burgdorferi* B31 temperature-shifted to 33°C or 37°C with no protein or antibody (negative control; “Unstained”) secondary antibody alone (negative control; “Ab only”), 0.25 nM Fc-tagged EGF (light blue), or 1 nM of Fc-tagged EGF (dark blue, red). Binding was determined by an Alexa Fluor 488-conjugated anti-Fc antibody. **(C)** PCA plot of *B. burgdorferi* treated with Fc-tagged EGF or the Fc tag alone at 33°C and 37°C shows treatment specific clustering of gene expression profiles. Individual samples are colored according to the legend at right, and colored ovals illustrate sample groupings. **(D)** A hierarchical clustering heatmap illustrates *B. burgdorferi* samples and genes differentially expressed by *B. burgdorferi* treated with EGF-Fc or the Fc tag alone at 37°C. Colors correspond with the Z-score per the legend below. Differentially expressed genes (DEGs) were identified by DESeq2 on Partek flow with default conditions, with a fold change threshold of ±2 (EGF/Fc) and p-values < 0.05. Below, gene set enrichment analyses comparing genes up or downregulated by EGF treatment at 37°C uncovered that EGF treatment significantly upregulated *B. burgdorferi* genes expressed by spirochetes in dialysis membrane chambers (“DMC”) and downregulated genes expressed by spirochetes in feeding nymphs ( “Nymph”) ^38^. Open circles indicate no significant enrichment, while closed circles indicate significance (p < 0.05).

We next sought to determine the validity of these screens by testing a selection of identified putative interactions using orthogonal methods. We thus used cell-based ELISA and flow cytometric assays to test interactions identified by screens with Lyme *Borrelia*. By ELISA, we validated that interleukins 28a and 29 (IL28a and IL29) and B- and T-Lymphocyte Attenuator (BTLA), bound to *B. burgdorferi* B31 lysates in a dose-dependent manner at levels over 50-fold greater than an irrelevant negative control protein, NeSt1 (^24^; Fig. 1F, p-values = 0.001, 0.002, and < 0.0001 for IL28a, IL29, and BTLA, respectively). Similarly, leukocyte peptidase inhibitor (SLPI) also bound *B. burgdorferi* B31 lysate in a dose-dependent manner as measured by ELISA in a separate experiment (Fig. S3C). Flow cytometric assays recapitulated binding by IL28a, IL29, and BTLA to *B. burgdorferi* (Fig. S3D), while a separate flow cytometric experiment validated *B. burgdorferi* binding by Galectin-3 (LGALS3) and Leukocyte cell-derived chemotaxin 2 (LECT2) (Fig. 1G). However, some BASEHIT-predicted interactions did not validate, for example, the predicted interactions between *B. burgdorferi* and Leukocyte Associated Immunoglobulin Like Receptor 1 (LAIR1; Fig. 1F, S3D), complement factor D (not shown), and AXL (Fig. 1G). Nevertheless, 9/12 of our attempted validations were successful (Shown in Fig. 1F-G, 2D-E, 3B, and S3C-D). This underscores the validity of our screens as 75% of the small subset of tested BASEHIT-predicted interactions were recapitulated in at least one orthogonal binding assay. That some BASEHIT interactions did not orthogonally validate may indicate the potential for false positives in the screen, though it is also possible that some of these interactions are valid, but that BASEHIT was more sensitive than the orthogonal assays, as has been found with this library previously ^19^.

### Analysis of hits among select pathogens uncovers diverse mechanisms of pathogenicity

We next conducted enrichment analyses to identify biological pathways significantly enriched by intracellular bacteria (Table S2; Fig. S3E). For example, proteins involved in protein folding and the response to endoplasmic reticulum stress were significantly enriched by screening with the Rickettsial pathogens *A. phagocytophilum*, *Rickettsia* spp., and *O. tsutsugamushi* (Fig. S3E). Proteins involved in signal transduction, organism development, and morphogenesis were significantly enriched in screens with *Rickettsia* samples (Fig. S3E). For example, *Rickettsia* bound fibroblast growth factor 1 (FGF1), whose receptor, FGFR1, is a known *Rickettsia* ligand ^25^. Samples of another Rickettsial pathogen, *O. tsutsugamushi*, bound to 67 unique proteins, including CXCL9, CXCR4, and CD68 (Table S2).

We also analyzed exoprotein binding patterns for the parasites *Babesia duncani*, sporozoites, trophozoites, and gametocytes of the human malaria parasite, *P. falciparum*, and sporozoites of the rodent malaria parasite, *P. berghei* (Table S2; Fig. S3E). Sporozoites are transmitted from mosquitos to the host, where they infect liver cells. Trophozoites give rise to merozoites within the red blood cells (RBCs), and gametocytes initiate the sexual infection cycle ^26,27^. Pellet and supernatant fractions from sporozoite purifications and healthy RBCs processed identically to *B. duncani* or to *P. falciparum* gametocytes and trophozoites were included as controls. Hits observed in matched controls were excluded from the list of hits for each corresponding life stage (Table S2). Proteins identified by screens with sporozoite samples were primarily involved in the regulation of the ERK1/ERK2 cascade (Fig. S3E), and many of the hits are correlated with malaria infection, resistance, or disease severity in mice and humans ^28–30^. For example, phospholipase A2 Group IIA (PLA2G2A) can directly kill *Plasmodium* via the production of toxic lipids, and overexpression of this protein enhances malaria resistance in mice ^28^. Other sporozoite hits included CXCL9, and the previously identified interaction partner CD74 ^30^.

### Characterization of *Leptospira* and *Borrelia* hits highlights possible roles in pathogenesis and host adaptation

We screened 6 serovars of pathogenic and nonpathogenic *Leptospira*, a zoonotic pathogen that is a spirochete like vector-borne *Borrelia*. To increase antigenic variation and investigate host adaptation, we not only grew *Leptospira* traditionally (30°C without NaCl supplementation), but also at 37°C supplemented with 120 mM NaCl (Fig. 2A), which induces the production of host-adapted virulence factors ^31^. As expected, *in vitro* host-adapted *Leptospira* bound to significantly more human proteins than untreated *Leptospira* (Fig. 2B; mean = 42 and 28 hits per sample, respectively; p-value = 0.031). We compared the GO terms enriched by screens with treated or untreated *Leptospira,* and found many of the proteins uniquely bound by the host-adapted *Leptospira* are involved in ion transport or arterial blood pressure (Fig. 2C). This includes a range of transmembrane ion transporters and channels, as well as vasopressin (AVP). Severe leptospirosis often induces renal failure characterized by polyuria and decreases in urinary osmolality ^32^, which is consistent with what would be expected with electrolyte imbalance and decreased vasopressin function ^32^. Notably, this vasopressin interaction was validated by flow cytometry and ELISA (Fig. 2D,E).

We also screened 14 strains of Lyme *Borrelia*, encompassing *B. burgdorferi*, *B. afzelii*, *B. garinii*, and *B. bavariensis*. To some degree, each genospecies is correlated with a characteristic Lyme pathology such as arthritis (*B. burgdorferi*) and neuroborreliosis (*B. bavariensis*) ^33–35^. Interestingly, *B. burgdorferi* uniquely bound a protein associated with rheumatoid arthritis (PGLRP1 ^16^), and *B. bavariensis* uniquely bound several proteins found in the central nervous system (FGF17 and multiple neuropeptides; Table S2).

Further, we compared hits of screens with *Borrelia* that had been shifted to temperatures to induce *Borrelia* protein expression similar to tick-adapted (23°C), host-adapted (37°C), and intermediary (26–33°C) states ^36,37^. As expected, we observed substantial variation in the hits and pathways enriched by *Borrelia* shifted to different temperatures (Fig. 3A). Hits unique to non-host-adapted spirochetes—those shifted to 23–33°C—enriched GO terms including antigen processing and presentation, lipid digestion, and regulation of the ERK1/2 response (Fig. 3A). Host-adapted *Borrelia* (shifted to 37°C) uniquely bound epidermal growth factor, and other proteins involved in EGF-related pathways (Fig. 3A; Table S2). We further validated this EGF-*Borrelia* interaction by flow cytometry, and found Fc-tagged EGF bound to *B. burgdorferi* grown at 33°C in a dose-dependent manner, with increased fluorescence of spirochetes incubated with 0.25 nM and 1 nM EGF. Host-adapted *B. burgdorferi* (those shifted to 37°C) incubated with 0.25 nM EGF exhibited a further shift in fluorescence beyond all other EGF-incubated spirochetes and controls (Fig. 3B). This result was concordant with the treatment-specific EGF binding activity seen with host-adapted spirochetes in the BASEHIT screens, and indicates that the EGF binding activity is dependent on the *Borrelia* growth temperature—and likely changes in *Borrelia* protein expression.

We next examined potential functional significance of the EGF:*B. burgdorferi* interaction *in vitro*. We first examined whether EGF affected *B. burgdorferi* growth and viability, but found that even daily treatment with 10 nM EGF had no impact on spirochete growth (Fig. S4A). As EGF binding appears to be influenced by *B. burgdorferi* host-adaptive gene expression, we next sought to determine if there is a reciprocal relationship by which *B. burgdorferi* gene expression is influenced by EGF binding. Indeed, we found that EGF-Fc treatment did impact gene expression at 37°C compared to treatment with Fc alone, and that these differences resulted in distinct treatment-specific groupings by PCA (Fig. 3C). Using a threshold of ± 2-fold enrichment, EGF treatment significantly upregulated 73 genes and downregulated 28 genes at 37°C (Fig. 3D). Further, EGF had minimal impact on gene expression in 33°C cultured spirochetes, with only 9 upregulated genes and 14 downregulated genes (Table S3). This indicates that the EGF-mediated gene expression is consistent with binding phenotypes, as 37°C treatment enhances both EGF binding and EGF-mediated gene expression.

Finally, we examined what genes are differentially regulated following EGF treatment. To survive in an unfed tick, during tick-to-host transmission, and during disseminated host infection, *B. burgdorferi* alter their gene expression to produce proteins specific to these diverse stages of its life cycle. The gene expression differences between tick-to-host transmission and disseminated infection have been highlighted recently in a study comparing the transcriptomes of *B. burgdorferi* in feeding nymphs (transmission) and dialysis membrane chambers implanted in rats (DMCs; mimicking disseminated infection) ^38^. We used the databases provided by this study to examine patterns of gene enrichment seen in EGF-treated spirochetes, and found that EGF treatment at 37°C, and to a lesser extent at 33°C, significantly enriched genes that are upregulated in DMCs (Fig. 3D, S4B, p-values < 0.0001 and = 0.04 for 37°C and 33°C-shifted spirochetes, respectively). Conversely, EGF-treated spirochetes at 37°C significantly downregulated genes that are expressed by spirochetes in feeding nymphs (Fig. 3D, p-value = 0.03). This was not seen at 33°C, consistent with the minimal changes to the gene expression profile, and lesser EGF binding at this temperature. Overall, these findings lead to the possibility that EGF is an environmental signal recognized by *B. burgdorferi* to coordinate stage-specific transcriptional regulation.

### Analysis of hits by pathogen niche reveal conserved nodes of interaction

**Figure 4.**
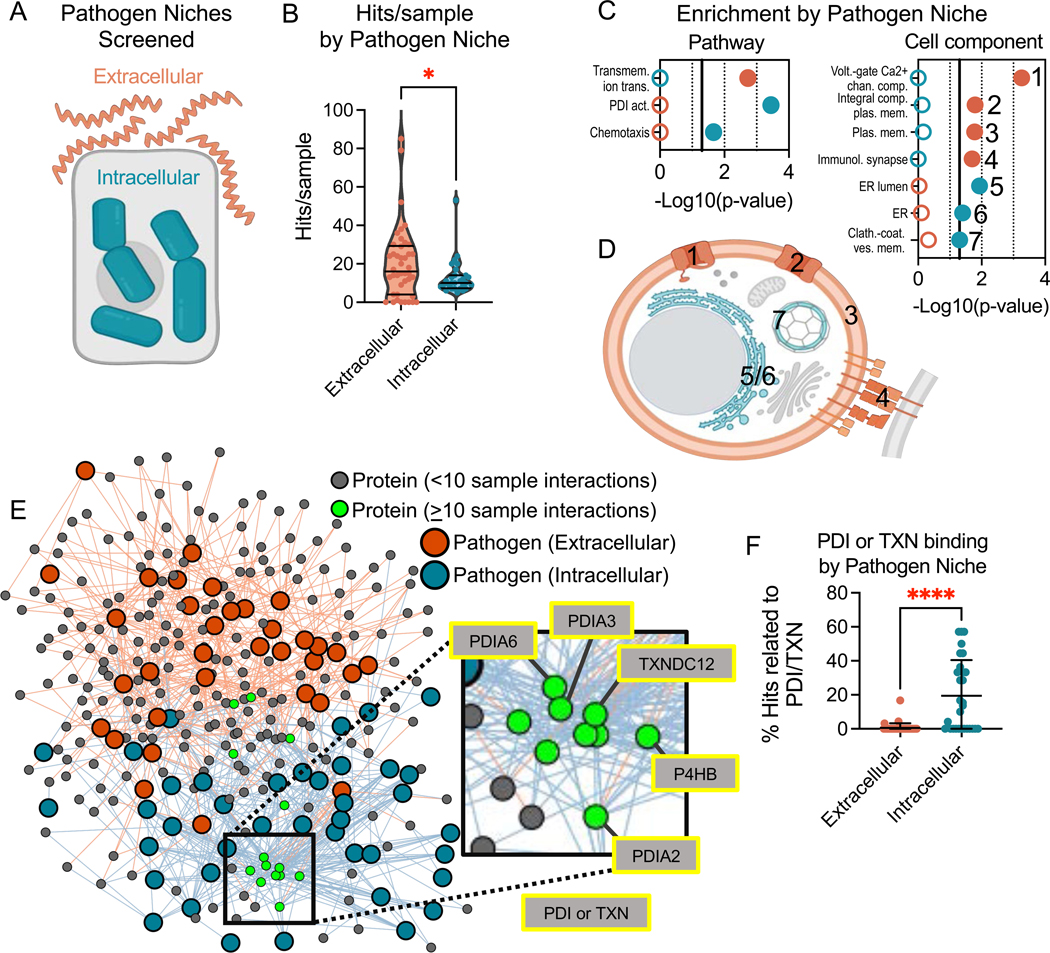
Comparison of intracellular and extracellular pathogen protein binding characteristics. **(A)** Hits from screens with extracellular (orange) and intracellular (blue) pathogens are depicted. **(B)** The number of hits for each extracellular intracellular pathogen sample are shown with a violin plot showing the mean and quartiles. Hits are defined as human proteins with normalized enrichment scores above pathogen-specific thresholds in at least 2 of 3 replicates. Extracellular pathogens bound to significantly more hits-per-sample than intracellular (Two-tailed Mann-Whitney test, p-value = 0.0004). **(C)** Gene ontology annotated biological pathways or cellular components that are significantly enriched among human proteins binding to extracellular or intracellular pathogens are plotted against their -Log_10_(p-value). Significant enrichment (beyond the solid vertical line at 1.3) is indicated by solid circles, while empty circles indicate no significant enrichment. Significance was determined by DAVID (EASE p-value < 0.05, κ > 0.5). **(D)** An illustration highlights that extracellular and intracellular pathogens hits enriched extracellular and intracellular cell components. Numbers correspond with those shown in **(C)**. **(E)** A global network analysis illustrating proteins (gray and green circles) interacting with at least two extracellular or intracellular samples are shown. Proteins shown to bind to at least 10 samples are highlighted in green, and the inset details the proteins making up a cluster of 9 such proteins displaying a strong bias for intracellular pathogen binding. Protein disulfide isomerases (PDI) are highlighted in yellow **(F)** The percentage of overall hits per sample that are PDIs or Thioredoxins (TXN) are shown for intracellular and extracellular pathogens. As expected, intracellular pathogens bound significantly more of these proteins than extracellular pathogens (Mann-Whitney test, p-value < 0.0001).

We next compared the binding patterns of intracellular versus extracellular pathogens (Fig. 4A). Given that the BASEHIT library was designed to include proteins with extracellular localization, we expected that extracellular pathogens would bind to more proteins in the library than intracellular pathogens. Indeed, we found that extracellular pathogens exhibited nearly twice as many hits-per-sample compared to intracellular pathogens, with means of 20 and 13 hits, respectively (Fig. 4B, p-value = 0.031). However, some proteins in the library can also be found intracellularly due to moonlighting—whereby a single protein exhibits multiple functions is found in multiple subcellular localizations (*e.g.*, the endoplasmic reticulum lumen and plasma membrane) ^39,40^. Thus, we tested whether intracellular functions or locales were significantly enriched among the intracellular pathogen hits. Comparisons of the GO Terms enriched by hits from intracellular and extracellular pathogens revealed that extracellular bacteria were enriched for interactions with proteins involved in transmembrane transport, and other transmembrane proteins (Fig. 4C, illustrated Fig. 4D). By contrast, intracellular pathogens interacted with proteins involved in protein disulfide isomerase (PDI) activity and chemotaxis, as well as other proteins found in the endoplasmic reticulum and clatherin-coated vesicles (Fig. 4C, illustrated Fig. 4D).

Network visualizations based on exoprotein interactions revealed that pathogens clustered together by their intra- and extracellular niche, further illustrating the clear dichotomy in protein binding profiles of pathogens with differing lifestyles (Fig. 4E). We next sought to determine which specific proteins preferentially bound to extracellular versus intracellular pathogens. Overall, 14 proteins bound to 10 or more samples (Fig. 4E). Four of these proteins bound to equal numbers of extra- and intracellular pathogens. However, the other 10 proteins displayed a clear bias for intracellular pathogens, and none of these 10 interacted with more than one extracellular sample. Interestingly, 6 of these 10 proteins were members of the protein disulfide isomerase (PDI) or thioredoxin (TXN) protein family (Fig. 4E insert). While PDI binding has previously been reported for a limited number of specific pathogens ^41–44^ where it is required for their ability to invade cells ^45,46^, we found that this feature was far more common: 78% of intracellular pathogens tested bound at least one PDI family member, compared to 15% of extracellular strains (Fig. 4F). These results indicate that PDI-mediated cell invasion may be a highly prevalent feature of intracellular pathogens.

### PDI inhibition limits cell invasion by Rickettsial pathogens

**Figure 5.**
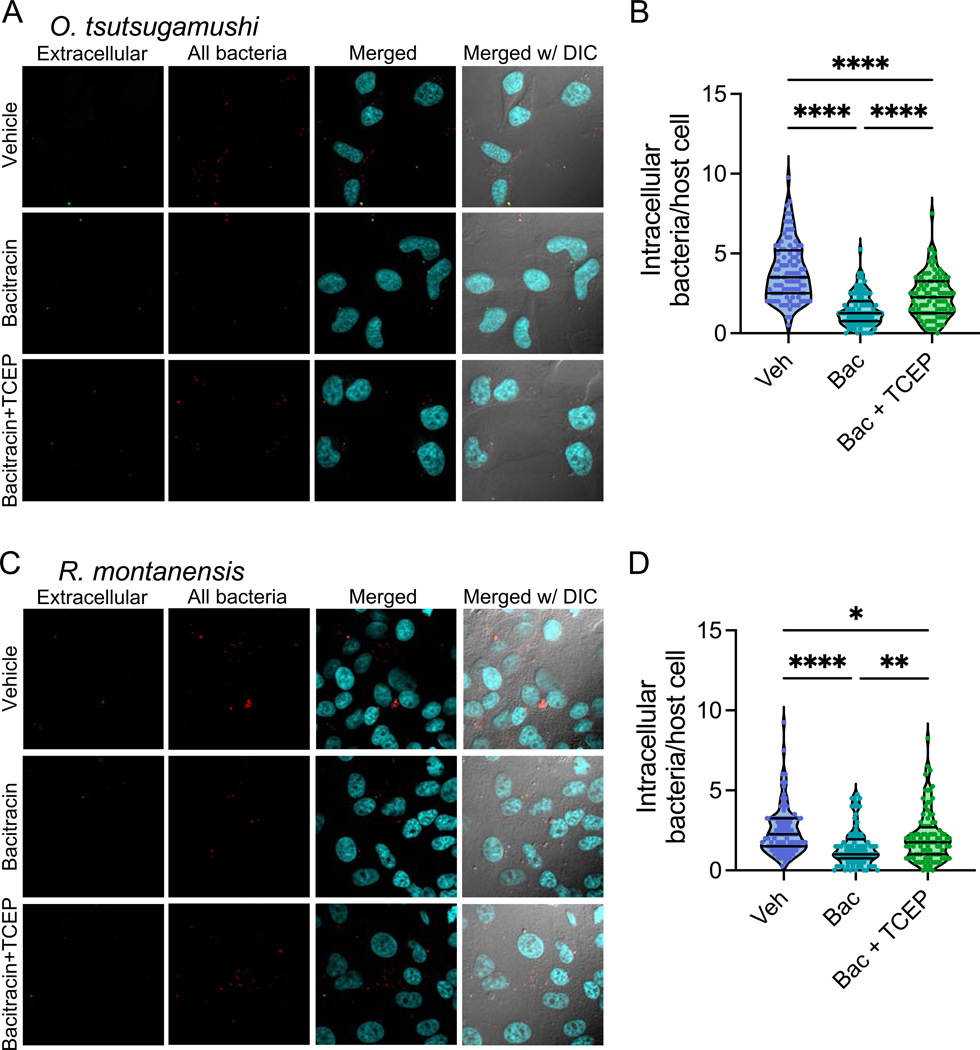
Bacitracin inhibition of intracellular pathogen cell invasion. **(A,C)** Cells were first treated with water (“Vehicle”) or bacitracin, then washed and incubated with *O. tsutsugamushi* or *R. montanensis* in the presence of water or a reducing agent, tris(2-carboxyethyl)phosphine (“TCEP”). Extracellular bacteria were then labeled with membrane impermeable Alexafluor-488 tagged antibodies, cells were permeabilized, then all bacteria (intracellular and extracellular) were labeled with Alexa Fluor-594 conjugated antibodies. Shown are representative images of **(A)** HeLa cells seeded with *O. tsutsugamushi* and **(C)** Vero76 cells seeded with *R. montanensis*. The intracellular bacteria per cell are shown as a composite of 4 experiments with 100 cells counted per experiment in **(B)** *O. tsutsugamushi* and **(D)** *R. montanensis* per cell were counted, and counts were compared across treatments (water: Blue, “Veh”, Bacitracin: Teal, “Bac”, and Bacitracin + TCEP: Green, “Bac + TCEP”). Comparisons were made between groups by Kruskal-Wallis test with a Dunn’s multiple comparisons test (**** = p-values <0.0001, ** = 0.0019, * = 0.0399.)

We previously showed that cell invasion by *A. phagocytophilum* and *E. chaffeensis* is facilitated by host PDIs ^45,46^. As such, we used a PDI-inhibiting membrane-impermeable antibiotic mix, bacitracin ^45,46^ to investigate whether PDI-mediated cell invasion is a conserved means of pathogen invasion using two BASEHIT-identified PDI-binding Rickettsial pathogens, *O. tsutsugamushi* and *R. montanensis*. Bacitracin-treated cells contained significantly fewer intracellular bacteria than the vehicle control-treated cells (Fig. 5A-D; means = 1.5 *O. tsutsugamushi* and 1.4 *R. montanensis* per bacitracin-treated cell, and 3.9 *O. tsutsugamushi* and 2.5 *R. montanensis* per vehicle-treated cell; p-values < 0.0001). These findings demonstrate that bacitracin inhibits cell invasion by these intracellular bacteria (Fig. 5A-D) despite demonstrating no direct bactericidal effect (Fig. S4C,D). To determine whether bacitracin-induced inhibition of cell surface reductases is responsible for inhibiting cell invasion ^47^, we next infected bacitracin-treated cells in the presence or absence of a cell-impermeable disulfide reducing agent (Tris(2-carboxyethyl)phosphine–HCl; TCEP) ^45,48^. Indeed, TCEP partially recovered cell invasion, with significantly more intracellular bacteria per cell than those treated with bacitracin alone (means = 2.3 *O. tsutsugamushi* and 2.1 *R. montanensis* per bacitracin-treated cell inoculated with TCEP; Fig. 5A-D; p-values = 0.005 and < 0.0001 for *O. tsutsugamushi* and *R. montanensis*, respectively). These results indicate that cell surface disulfide reductases facilitate cell invasion for *O. tsutsugamushi* and *R. montanensis*, and that targeting host PDIs limits cell invasion by these intracellular pathogens.

## DISCUSSION

Here, we comprehensively mapped host-pathogen interactions across the human exoproteome. This resulted in an atlas of 1,303 interactions between 713 unique human exoproteins and 82 bacterial and parasite samples from dozens of vector-borne and related pathogen species. Analyses of the proteins bound by different microbes uncovered unexpected molecular mechanisms that may drive pathogen-specific disease manifestations. Our validation and functional comparison efforts provided clear logical grounding to support the validity of the BASEHIT data, and highlighted host-pathogen interactions that may serve as valuable targets for interventions and therapeutics.

Our pathogen BASEHIT screens uncovered twice as many binding partners per sample than our recent screens with human commensal microbiota samples ^18^. This is likely because the evolutionary relationship between humans and their commensal microbes is often different from pathogens. For example, humans and their microbiomes have co-evolved to form a frequently symbiotic relationship that is often biased toward immune tolerance ^49,50^, and indigenous microbes typically inhabit a relatively narrow range of tissues (*e.g.*, the gastrointestinal tract, skin, etc.) ^51,52^. The human-pathogen relationship, on the other hand, has developed primarily through an evolutionary arms race, whereby the host has evolved mechanisms of pathogen clearance (such as immune cell receptors and antimicrobial peptides), and pathogens have evolved myriad immune evasion techniques (such as atypical PAMPs and recruitment of complement inhibitors) ^5–9,11,12^. Further, many pathogens infect and disseminate to multiple tissues, requiring the ability to bind many different ECM and cell surface components ^1–4,53–55^. Because of these contrasting evolutionary relationships, pathogens may bind to or be recognized by many more human proteins than endogenous microbiota samples. Indeed, in our studies with commensal bacteria, we found that bacteria that were annotated via ProTraits as ‘pathogenic in mammals’ were significantly enriched in host protein binding compared to other commensals ^18^.

In some cases, our analysis uncovered many predicted pathogen-human interactions for some organisms. In the most striking example, we identified 317 unique host interacting partners of Lyme *Borrelia*. These spirochetes are known to be recognized by many immune system molecules, recruit a wide variety of immune system inhibitors, and bind numerous adhesion receptors and ECM components to facilitate infection, dissemination, and colonization of a wide range of tissue types ^56,57^. Consistent with their considerable host interaction capabilities, *Borrelia* express over 150 outer surface proteins throughout their disease cycle ^23,56–60^. BASEHIT thus provides a detailed accounting for the host side of *Borrelia’s* interaction network, though additional work is needed to validate these interactions and identify their corresponding microbial binding partners.

The analyses of bound proteins also uncovered multiple intriguing potential mechanisms of disease pathogenesis. For example, we screened *O. tsutsugamushi*, the intracellular agent of scrub typhus. Given that these bacteria infect humans in an extracellular form and quickly invade human cells ^61^, we screened them in both their extracellular and intracellular forms. We found that extracellular, but not intracellular, *O. tsutsugamushi* bound to CD68, a protein highly expressed by monocytes and macrophages ^62^. Importantly, *O. tsutsugamushi* co-localizes with CD68^+^ monocytes and macrophages, possibly infecting these cells as a means of dissemination ^63^. The results presented here indicate a direct interaction between CD68 and *O. tsutsugamushi*, leading to the possibility that this may be a mechanism that mediates CD68^+^ cell invasion. Furthermore, *O. tsutsugamushi* also bound to a widely expressed chemokine receptor, CXCR4 ^64,65^, which is used by HIV to infect naïve CD4^+^ T cells via the viral protein gp120 ^66^. Interestingly, an *O. tsutsugamushi* surface-bound protease has been found to share a high degree of homology with the CXCR4-binding region of gp120 and *O. tsutsugamushi* elicits cross-protective immunity to HIV by interfering with gp120’s CXCR4 binding ^66^. Here we describe the potential direct binding of *O. tsutsugamushi* to CXCR4 and suggests a potential role for this receptor in scrub typhus pathogenicity, as well as additional mechanisms whereby *O. tsutsugamushi* may impact HIV co-infection.

Our work also demonstrated that *Plasmodium* sporozoites, gametocytes, and trophozoites bound to a wide range of proteins involved in cytokine responses. It has long been thought that an overexuberant immune response is in part responsible for the pathogenesis of malaria ^67–69^, and some of the proteins bound by our *Plasmodium* samples are correlated with severe malaria. For example, sporozoites bound to chemokines including CXCL9 and the receptor CD74, though these interactions were not observed in gametocyte and trophozoite screens. Previous work has uncovered the CD74 interaction, and the identified *Plasmodium* ligand is produced in sporozoites and other stages within the host ^30^. An interaction with CXCL9 has not been previously reported, though this may occur through a similarly ubiquitously expressed ligand. CXCL9 is upregulated in the brains of mice with experimental cerebral malaria, and mice deficient in CXCL9 do not develop cerebral malaria ^29^. This suggests the possibility that the identified *Plasmodium*-CXCL9 binding may contribute to cerebral malaria due to immune dysregulation. We also identified *P. falciparum* gametocytes bound to the cytokine receptor IL-15Rα. IL-15 has been shown to facilitate parasite clearance via a Type I immune response, and treatment with IL-15 has been shown to protect mice against cerebral malaria ^70^. This raises the possibility that *Plasmodium* may bind the IL-15 receptor to interfere with IL-15-mediated immune responses and evade clearance from the host. The impairment of *Plasmodium* clearance and immune dysregulation in this way could contribute to more severe complications.

We screened multiple strains of *L. interrogans* and *L. biflexa* with and without NaCl treatment to stimulate the production of host-adaptive virulence factors. *Leptospira* are found environmentally in low osmolar environments, such as soil and water, and transition to the higher osmolar host environment upon infection. The spirochetes produce different outer surface proteins in response to their environment, and NaCl treatment shifts the antigenic profile to a more host-adapted state during *in vitro* growth ^31^. We found that NaCl-treated *Leptospira* interacted with many unique proteins, including the antidiuretic hormone vasopressin and one of its receptors. Interestingly, one manifestation of severe Leptospirosis is renal failure accompanied by increased urine volume and decreased urine osmolality—symptoms that are consistent with an impaired activity of vasopressin ^32^. In fact, a glycolipoprotein isolated from pathogenic, but not nonpathogenic, *Leptospira* blocks vasopressin activity in an *in vitro* model of guinea pig kidneys ^71^. We found that *L. interrogans* bound to vasopressin in a dose-dependent manner. We show a direct interaction between pathogenic *Leptospira* and vasopressin, implicating this as a potential molecular mechanism of severe leptospirosis, and suggesting potential avenues for leptospirosis treatments.

Comparisons of screens with various genospecies of Lyme *Borrelia* demonstrated that strains of the genospecies *B. bavariensis*—which is associated with neurological manifestations of Lyme disease ^35^—uniquely bound to many proteins found in the brain and central nervous system. Given that *B. bavariensis* infection often leads to neuroborreliosis, these brain-specific interactions may be mechanisms of pathogenesis. We also compared screens of *B. burgdorferi* exposed to various culture temperatures, and found that *Borrelia* exposed to 37°C (mimicking the host environment) uniquely bound to proteins related to epidermal growth factor (EGF) signaling, including EGF itself. Further, we found that EGF treatment induces gene expression changes in *B. burgdorferi* reminiscent of later stages of host infection ^38^. While environmental factors such as temperature, pH, and nutrients have all been shown to induce changes to *B. burgdorferi* gene expression, now we have found transcriptional changes due to the direct binding of a host protein ^23,72,73^. Furthermore, since this interaction and the resulting gene regulation is enhanced by shifting *B. burgdorferi* to 37°C, one could hypothesize that host-adapted *B. burgdorferi* produce a specific EGF receptor to coordinate this stage-specific gene expression.

We observed widespread PDI binding by intracellular pathogens, which implicates these interactions as potential targets for broadly effective therapeutics for diverse human infectious diseases. Though frequently thought of as endoplasmic reticulum-resident proteins, PDIs are also secreted by a wide variety of cell types ^41^. Recruitment of these extracellular PDIs has been demonstrated to be essential for cell invasion by intracellular pathogens including *A. phagocytophilum* and *E. chaffeensis* ^45,46^, as well as several viruses ^74–76^. Here, we observed previously characterized PDI interactions ^45^, and observed previously undescribed PDI binding by *O. tsutsugamushi*, *Rickettsia* spp., and *B. duncani*. Further, we show that *O. tsutsugamushi* and *R. montanensis* use PDIs as a method of cell invasion. Cell adhesion and invasion by *O. tsutsugamushi* and *R. montanensis* is multifactorial ^77–83^, leading to robust albeit incomplete inhibition of invasion by bacitracin (and rescue by TCEP) seen here. However, this degree of inhibition mirrors what we have seen with other Rickettsial pathogens ^45,46^. When viewed in light of our BASEHIT screening results, this indicates that PDI engagement may be a generalized strategy for host cell invasion for a wide range of intracellular pathogens. As such, therapeutics targeting interactions with host PDIs—as opposed to targeting the microbes themselves—could represent a treatment strategy for patients infected by PDI-interacting intracellular pathogens. While adverse effects of non-selective PDI inhibitors preclude their use as therapeutics ^84,85^, targeted PDI inhibitors have been generated and are well-tolerated preclinically ^85,86^. Selective PDI inhibitors that target the specific secreted PDIs engaged by pathogens may represent effective and broad-spectrum antibiotics that are less susceptible to antibiotic resistance.

Taken together, this study provides a foundational database of human-pathogen interactions to support a wide range of future studies investigating the evolutionary and molecular mechanisms of vector-borne disease pathogenesis. Future work may focus on interactions that are conserved among many pathogens, such as PDIs and intracellular pathogens. These studies may detail the evolutionary pressures leading to such convergent evolution or serve as the basis for comparative genomics of the pathogens to identify conserved motifs facilitating such interactions. These and other insights may enable identification of urgently needed anti-microbial agents and mechanistically-tailored therapeutics for vector-borne pathogens.

### Limitations of the study

The screens presented here have important limitations. First, BASEHIT screens can only capture interactions between pathogens and individual human proteins, thus any interactions that require a human protein complex cannot be observed. Similarly, many known interactions were not observed in our screens, indicating that some members of the library were not properly folded on the yeast cell surface. Alternatively, it may be that the culture conditions used did not elicit the full spectrum of binding partners expressed on the pathogens for host protein interaction. This point is underscored by the observed impacts of culture osmolarity and temperature on BASEHIT results for *Leptospira* and *Borrelia*. Finally, we orthogonally validated and functionally characterized a relatively small subset of the detected host protein-pathogen pairings. Additional study is thus warranted to biophysically validate and determine the functional impact of a larger portion of the BASEHIT interactions.

## STAR Methods

### RESOURCE AVAILABILITY

#### Lead Contact

Further information and requests for resources and reagents should be directed to and will be fulfilled by the lead contact, Aaron M. Ring (aaronring@fredhutch.org).

#### Materials availability

This study did not generate new unique reagents.

#### Data and code availability

- Pathogen-protein interaction data and *Borrelia burgdorferi* gene expression data are deposited in supplemental tables, and any additional data reported in this paper will be shared by the lead contact upon request.
- This paper does not report original code.
- Any additional information required to reanalyze the data reported in this paper is available from the lead contact upon request.

### EXPERIMENTAL MODEL AND STUDY PARTICIPANT DETAILS

#### Microbe strains

##### Borrelia burgdorferi sensu lato.

14 isolates of Lyme *Borrelia* were screened, including *B. burgdorferi sensu stricto* strains N40 (NCBI: txid521007), B31 (NCBI: txid224326), HP19 ^87^, CA8 (NCBI: txid747491), CT-1 ^88^, VS134 ^89^, and NT-1 ^90^, *B. afzelii* strains CB43 ^91^, TV34, and TV38, *B. garinii* strains G25 ^92^ and TV39, and *B. bavariensis* strains PBi (NCBI: txid290434) and PNi ^93^ (Table 1). These were grown at 33°C in Barbour-Stoenner-Kelly H (BSK-H) complete medium with 6% rabbit serum (Sigma; B8291–100ML). *Borrelia* were grown to approximately 5 × 10^6^ or 5 × 10^7^ cells/ml, then placed at 33°C or 37°C, or at 23°C, 26°C, or 30°C, respectively, for 24 hours, as indicated in Table 1. *Borrelia* were then heat-inactivated at 56°C for 30 minutes and harvested at 6,000 *g*. A total of 5 × 10^8^ cells were harvested for biotinylation and BASEHIT screens (Described below).

##### Leptospira.

Pathogenic *L. interrogans* serovars Manilae L495 (NCBI:txid214675), Copenhageni L1–130 (NCBI:txid267671), Canicola (NCBI:txid211880), and Lai 56601 (NCBI:txid189518), *L. licerasiae* Varillal VAR010 (NCBI:txid1049972), and nonpathogenic *L. biflexa* serovar Patoc (NCBI:txid145259; Table 1) were maintained in semi-solid Ellinghausen–McCullough–Johnson–Harris (EMJH) media (BD Biosciences; 279410) at 30°C ^94^. *Leptospira* were grown under conditions mimicking an aspect of the *in vivo* host environment known to induce virulence gene expression *in vitro* ^31^: Briefly, mid-logarithmic cultures were harvested by centrifugation at 12,000 *g.* Pelleted cells were washed twice with 1X phosphate buffered saline (PBS), resuspended in liquid EMJH medium supplemented with 120 mM NaCl, and then incubated at 37°C for 4 hours. Untreated *Leptospira* for controls were incubated at 30°C for 4 hours. Following treatment, *Leptospira* were heat-inactivated at 56°C for 30 minutes, and 10^9^ cells were harvested for BASEHIT screens.

##### Plasmodium berghei.

*P. berghei* ANKA GFPcon 259cl2 (ATCC; MRA-865)-infected *Anopheles gambiae* 4ARR (ATCC; MRA-121) mosquitos were produced by feeding on infected Swiss Webster mice as described previously ^95^. 17–21 days after feeding, midguts and salivary glands from approximately 50 infected mosquitos were separated and dissected, and sporozoites were isolated as previously described ^96,97^. Briefly, isolated midguts and salivary glands were washed in PBS and repeatedly passed through 28 ½-gauge insulin syringes to release the sporozoites. The mixture was layered on a 17% w/v solution of Accudenz (Accurate Chemical; AN7050) and then centrifuged at 2,500 *g* for 20 minutes to separate sporozoites from mosquito tissues ^97^. Approximately 10^6^ sporozoites were isolated from each tissue, and the sporozoite-depleted initial debris pellet and the final supernatant were collected as negative controls for BASEHIT screens.

##### *Plasmodium falciparum in vitro* culture.

*P. falciparum* NF54 (NCBI:txid5843) gametocytes and trophozoites were cultured as previously described ^98,99^. Briefly, *P. falciparum* was grown in O^+^ red blood cells at 4% hematocrit and RPMI 1640 medium (Corning; 10–041-CV) supplemented with 10 mM glutamine, 25 mM HEPES, 50 μg/ml hypoxanthine and 10% O^+^ human serum. Parasite cultures were maintained in a candle jar at 37 °C to provide a microaerophilic environment. To isolate trophozoite stage of the parasite, cultures were synchronized by treatment with 5% sorbitol as described earlier ^100^, followed by culture for another 72 hours. For gametocytes, parasite culture was initiated with 0.5% low passage asexual stage culture and maintained for 9 and 15 days with daily media changes without addition on new RBCs. Gametocyte cultures were treated with 50mM N-acetylglucosamine for 72 hours to clear residual asexual stages ^99^. Infected erythrocytes were then enriched by magnetic columns (25 LD columns, Miltenyi Biotec; 130–042-901) as previously described ^101^. Approximately 10^8^ infected erythrocytes were collected from each group. Uninfected erythrocytes treated identically for 15 days and collected by centrifugation were included as negative controls. *A. gambiae* Keele (NCBI:txid7165) or *A. stephensi* Liston strain (NCBI:txid30069) were infected with *P. falciparum* through a standard membrane feeding assay on an 18-day old *P. falciparum* gametocyte culture as previously described ^99^, and approximately 100 fed mosquitos of each species were collected. 18 days after feeding, sporozoites were isolated from salivary glands and midguts as described above, resulting in approximately 10^6^ sporozoites from each tissue. The debris and supernatant samples were collected for use as negative controls in BASEHIT screens.

##### Orientia tsutsugamushi.

*O. tsutsugamushi* strains TA768, Gilliam (NCBI:txid1359184), TA686, Ikeda (NCBI:txid334380), UT76 (NCBI:txid682184), Karp (NCBI:txid1359185), and Kato (NCBI:txid1359186) were grown on confluent monolayers of L929 cells (RRID: CVCL_0462) in Dulbecco’s Modified Eagle Medium (DMEM, ThermoFisher; 21013) supplemented with 10% heat-inactivated fetal bovine serum (FBS; Gibco; 16140071) at 37°C with 5% CO_2_ as previously described ^61^. Strains Ikeda and Karp were similarly cultivated in HeLa cells (RRID: CVCL_0030) cells as previously described ^102^. Extracellular and intracellular *O. tsutsugamushi* were separated as previously described ^61^ for all but *O. tsutsugamushi* Ikeda grown on HeLa cells. Briefly, cells and culture media were transferred to 50 ml conical tubes and cells were pelleted at low speed. To isolate extracellular *O. tsutsugamushi*, the supernatant was transferred to a clean tube, and *O. tsutsugamushi* were harvested by centrifugation at 20,000 g. To harvest the intracellular *O. tsutsugamushi*, the pelleted L929 cells were homogenized, and the resulting lysate was centrifuged at a low speed to remove host cell debris. The supernatant was then transferred to a clean tube, and the released *O. tsutsugamushi* were harvested by centrifugation at 20,000 g. Approximately 10^7^ - 10^9^ total cells of each strain and bacterial population (intracellular/extracellular) were collected, and heat inactivated at 56°C for 30 minutes. HeLa cells inoculated with the strain Ikeda were mechanically lysed using glass beads as described ^102^, and the resulting cell suspension was then passed through a 27-gauge blunt end needle five times. Lysates were centrifugated at 200 g for 5 minutes to remove cellular debris, and the supernatant was centrifuged at 2,739 g for 10 minutes to yield a partially purified bacterial cell pellet that was subsequently resuspended in ice-cold SPG buffer (0.22 M sucrose, 3.7 mM KH2PO4, 7.0 mM K2HPO4, 5 mM L-glutamine, pH 7.0). The SPG suspension was overlaid on a 25% Renografin cushion followed by centrifugation at 21,000 g at 4°C for 30 minutes in an Optima XE-90 ultracentrifuge (Beckman Coulter) using a SW 41 Ti Swinging-Bucket Rotor (Beckman Coulter). The remaining *O. tsutsugamushi* pellet was washed three times in ice-cold SPG after which the purified were biotinylated as described below.

##### Chlamydia trachomatis.

*C trachomatis* serovar L2 strain 434 (ATCC VR-902B; NCBI:txid471472), mycoplasma-free, was grown in HeLa cells in 1:1 DMEM:F12K (Sigma; D7777 and N3520) with 10% qualified FBS (ThermoFisher; 26140079) and 10 μg/ml cycloheximide (Sigma; 01810). For elementary body purification, HeLa cells ~ 40h post-infection were recovered using borosilicate beads, sonicated (QSonica model 500; high intensity ultrasonic water bath, 3 × 10 sec pulses at 40% amplitude), then *Chlamydia* elementary bodies purified using discontinuous gastrograffin (Bracco; 0270–0445-40) density gradients, at 40,000 g for 30 minutes, as described ^103^. Resulting stocks of purified elementary bodies were diluted in sucrose phosphate buffer (SPG) and 6 × 10^8^ elementary bodies were UV-inactivated with 10,000 × 100uJ/cm^2^ (Spectralinker XL-1000).

##### Babesia duncani.

*B. duncani* parasites (NCBI:txid323732) were cultured *in vitro* in human RBCs as reported earlier ^104^. The parasites were cultured in a complete HL-1 medium (Lonza; 344017) supplemented with 20% heat-inactivated FBS, 2% 50X HT Media Supplement Hybrid-MaxTM (Sigma; H0137), 1% 200 mM L-Glutamine (Gibco; 25030–081), 1% 100X Penicillin/Streptomycin (Gibco; 15240–062) and 1% 10 mg/ml Gentamicin (Gibco; 15710–072) in A^+^ RBCs at 5% hematocrit. The cultures were maintained at 37°C under a 2% O_2_/ 5% CO_2_/ 93% N_2_ atmosphere in a humidified chamber. Culture medium was changed daily and the parasitemia was monitored by examination of Giemsa-stained thin-blood smears using light microscopy. When parasitemia reached more than 20% and a large number of free merozoites were observed, the culture was centrifuged at 1800 rpm for 5 minutes. The culture supernatant containing the free merozoites was collected in a new tube and centrifuged at 4000 rpm for 10 minutes. The merozoite pellet was resuspended in 100 ml of complete HL-1 medium. Around 2–5 ml of merozoite suspension was used in hemocytometer to calculate the merozoite numbers in 100 ml. Approximately 10^9^ merozoites were collected.

##### Anaplasma phagocytophilum.

*A. phagocytophilum* strains HZ (NCBI:txid212042) and NCH1 (NCBI:txid1359161) were grown as previously described ^105^ in HL-60 cells (ATCC; CCL-240; RRID:CVCL_0002) at 37°C using RPMI medium (Thermo Fisher Scientific; 10–041-CV) supplemented with 10% FBS (GeminiBio; 100–106) and 1X Glutamax (ThermoFisher; 35050061). Bacterial numbers were calculated using the formula: number of infected HL-60 cells × 5 morulae/cell × 19 bacteria/cell × 0.5 (representing 50% recovery rate) ^106^, and the percentage of infection was measured using the Richard-Allan Scientific^™^ three-step staining (ThermoFisher; C997F97). Bacteria were purified by passing infected cells through a 27-G bent needle and using a series of centrifugation steps, as previously described ^105^. Approximately 6 × 10^8^ cells were isolated for BASEHIT screens.

##### Francisella tularensis.

*F. tularensis* live vaccine strain (LVS; NCBI:txid376619) was grown for 2 days at 37°C on Mueller Hinton plates, starting from glycerol stocks, and then collected and resuspended into Mueller Hinton broth, as previously described ^105^. Afterwards, the number of bacteria/ml was calculated by dilution series. Approximately 8 × 10^10^ bacteria were pelleted for BASEHIT screens.

##### Ehrlichia chaffeensis.

*E. chaffeensis* was cultured at 37°C in the canine macrophage cell line DH82 (ATCC; CRL-3590; RRID:CVCL_2018), using DMEM F12 (1:1) media (Thermo Fisher Scientific; A4192001) supplemented with 5% FBS (GeminiBio; 100–106) ^107^. Enumeration and isolation of bacteria was performed as described for *A. phagocytophilum*. Approximately 6 × 10^8^ cells were isolated for BASEHIT screens.

##### Rickettsia:

*R. typhi* strain Wilmington (NCBI:txid257363), *R. rickettsii* strain Sheila Smith (NCBI:txid392021), and *R. montanensis* strain M5/6 (NCBI:txid1359200) were grown as previously described ^108^. Briefly, *Rickettsia* were propagated in Vero 76 cells (African green monkey kidney; ATCC; CRL-1587; RRID:CVCL_0603) grown in DMEM (Mediatech, Inc.; 10–013-CV) supplemented with 5% FBS (GeminiBio; 100–106) at 34°C and 5% CO_2_. Cells were lysed by mild sonication for 5 seconds using a sonic dismembrator (FB505, Fisher Scientific). The cell lysate was filtered through a 5.0-μm pore-size filtering unit (Millipore). Approximately, 1 × 10^9^ Rickettsiae were collected by centrifugation.

### METHOD DETAILS

#### BASEHIT screening

##### Pathogen labeling.

Pathogens were biotinylated as previously described ^16–18^. Briefly, isolated pathogens were washed 3 times with 1 ml PBS, resuspended in 1 ml PBS with 5 μM Sulfo-NHS-LC-Biotin (ThermoFisher; A39257), and incubated at 37°C for 30 minutes. Samples were then treated with 10 μl of 1M Tris pH 8.0, washed with 1 ml PBS, and resuspended in 1 ml 10% glycerol in PBS (v/v) for storage at -80°C.

##### Yeast library screening.

The BASEHIT yeast display library was prepared for screening similar to previously described ^16–19,109^. Briefly, our pooled human ectodomain yeast display library was grown for 18 hours in SDO-Ura at 30°C and 225 RPM shaking to midlog phase, then resuspended in 90% SGO-Ura and 10% SDO-Ura to induce ectoprotein production for 18 hours at 30°C and 225 RPM shaking. Protein production was verified by flow cytometry using PE-conjugated antibodies recognizing a FLAG-epitope (BioLegend; 637309; RRID:AB_2563147) that was conjugated to the human ectodomains. Plasmid DNA was extracted from 400 μl of this induced yeast by Zymoprep Yeast cell Plasmid Miniprep II kits (Zymo Research; D2004) for a pre-selection screening control. The remaining yeast were then washed and resuspended in PBE (0.5% BSA, 0.5 mM EDTA, in PBS), and 3 × 10^7^ yeast were pelleted to the bottoms of 96-well v-bottom microtiter plates at 2,000 RPM for 5 minutes. The pelleted yeast were resuspended in 100 μl of PBE + 50 μl of an individual biotinylated pathogen sample. One well contained the entire pooled library and one pathogen sample, and each sample was run in triplicate. Three wells of yeast resuspended in 150 μl PBE but no pathogen were included in each plate as a negative control. The resuspended yeast were then incubated at 4°C with shaking for 2 hours. The yeast were then washed in PBE, resuspended in 100 μl of a 1:100 dilution of streptavidin-coated magnetic beads (Spherotech, 0.29 μm; SVM-025–5H) in PBE, and incubated for 1 hour at 4°C with shaking. Yeast were washed and resuspended in 100 μl PBE, and a custom 96-well magnet was used to select, wash, and elute bead-bound (and thus pathogen-bound) yeast. The magnet consists of a 3D printed body housing 96 1 cm magnetic posts. The posts were covered with a sterile PCR plate then submerged into the 96-well yeast cultures to select the streptavidin bead-coated pathogen-yeast complexes on the surface of the PCR plate. The magnet and PCR plate were then moved to a new 96-well microtiter plate containing 200 uL PBE, and the magnet was removed, and the PCR plate was agitated for 1 minute to allow the yeast to fall away. This was repeated for a total of 2 washes, then the same method was used to elute the yeast into SDO-Ura. The selected yeast were grown at 30°C with shaking for 48 hours for isolation of plasmids encoding the uniquely barcoded human proteins.

##### Library preparation and next generation sequencing.

Library preparation and sequencing was conducted as previously described ^18,19,109^. Plasmid DNA was extracted from the selected yeast using a Zymoprep-96 Yeast Plasmid Miniprep Kit (Zymo Research; D2007). An initial PCR to amplify the protein-producing clone-specific barcode was run with Phusion High-Fidelity DNA Polymerase (New England Biolabs) according to manufacturer’s conditions (58°C annealing, and 20 cycles of amplification). This PCR product was then used as the template for another PCR using Nextera i5 and i7 primers (Illumina) and the same conditions as the first PCR to index and prepare samples for multiplex sequencing. The PCR product was then pooled and run on a 2% agarose gel, and the 257 base pair product was cut and extracted using a Qiaquick Gel Extraction Kit (Qiagen; 28706). The resulting barcode library was then run on an Illumina MiSeq 150-Cycle Reagent Kit v3 (Illumina; MS-102–3001) according to manufacturer’s protocol, and a minimum of 0.1M reads per sample.

#### Validations

##### ELISA

###### Borrelia.

*B. burgdorferi* B31 lysate was generated using BugBuster reagent (Millipore Sigma; 70584–3) according to manufacturer’s instructions. 10 μg of the resulting lysate or 3% BSA (negative control) was then coated onto microtiter plate wells. Coated plates were then probed with 0.2 or 2 μg of His-tagged NEST1 (negative control ^110^), IL28a, IL29, BTLA, ULBP, and LAIR1 (R&D Systems; 1587-IL, 1588-IL, 9235-BT, 11196-UL, 2664-LR, respectively). A 1:20,000 dilution of HRP-conjugated Rabbit anti-His tag antibody (abcam; ab1187; RRID:AB_298652) was added to detect bound His-tagged proteins. Wells were then incubated with 100 μl of tetramethyl benzidine (TMB) solution for 12 minutes at room temperature, and the reaction was stopped with 100 μl of 0.5% hydrosulfuric acid. Plates were then read at a wavelength of 450 nm.

###### Leptospira.

Microtiter plates were coated with 1 or 100 ng of recombinant untagged vasopressin (AVP; R&D Systems; 2935). 10^8^ heat-inactivated *L. interrogans* serovar Copenhageni L1–130 were biotinylated in the same manner used for BASEHIT preparations and added to each well. 100 μl of HRP-conjugated streptavidin (1:500 dilution; ThermoFisher; N100) was then added to each well. Binding was determined by TMB-based colorimetry as done for *Borrelia*.

##### Flow Cytometry

###### Borrelia and Leptospira.

10^7^ *B. burgdorferi* N40, *L. interrogans* serovar Copenhageni, or *L. biflexa* serovar Patoc were washed with PBS, blocked for 1 hour with 3% BSA in PBS, then incubated for 1 hour for *Borrelia*: 2 μg of His-tagged IL28a, IL29, BTLA, or LAIR1, or 1 and 4 μg of Fc-tagged EGF (GenScript; Z03377) or 1 μg of an Fc isotype negative control, or for *Leptospira*: 0.1 and 2 μg of vasopressin. Spirochetes were then washed and fixed with 4% paraformaldehyde for 1 hour. Spirochetes were then probed with an anti-6XHis monoclonal antibody conjugated to Alexa Fluor 488 (for all but vasopressin: ThermoFisher, MA1–21315-A488; RRID:AB_2610645) and incubated for 1 hour, or, for vasopressin experiments, polyclonal rabbit anti-AVP antibody (ThermoFisher, PA5–112845; RRID:AB_2867579), followed by PE-conjugated anti-rabbit IgG antibody (ThermoFisher, 12–4739-81; RRID:AB_1210761) with 1-hour incubations each. Samples were run through an SA3800 Spectral Analyzer (Sony Biotechnology), and results were analyzed using FlowJo.

##### EGF Functional Validations with B. burgdorferi

###### B. burgdorferi growth with EGF treatment.

*B. burgdorferi* B31 were grown as above at 33°C or 37°C, and supplemented with 10 nM Fc-tagged EGF (GenScript; Z03377) or the Fc tag alone diluted in PBS every 24 hours for 7 days. Motile spirochetes were counted daily using an INCYTO C-chip disposable hemocytometer (INCYTO; DHC-N01).

###### Transcriptomic analysis of EGF-treated B. burgdorferi.

*Borrelia burgdorferi* B31 were grown to 1 × 10^7^ cells/ml as described above at 33°C. The cultures were then transferred to 37°C or maintained at 33°C for 24 hours. After 24 hours, 1nM Fc-tagged EGF (GenScript; Z03377) or the Fc tag alone were added, and cultures were left to incubate at the appropriate temperature for 24 more hours. Following this incubation, RNA was isolated by Monarch Total RNA Miniprep Kit (NEB; T2010S) and sequenced by Illumina NovaSeq 6000 with 100 base pair paired-end sequencing, with a minimum of 20 million reads per sample and a quality score of 35–36 per sample, according to manufacturer’s protocol. Significant differences in gene expression were then calculated by DESeq2, a 2-dimensional PCA was run, and a hierarchical clustering heatmap (with a threshold of ± 2-fold change in expression EGF/Fc at 37°C and p-value < 0.05) were all done using Partek Flow 10.0.23.0720 (Partek Incorporated). Genes upregulated in dialysis membrane chambers (disseminated host infection) or fed nymphs (tick-to-host transmission) were described in Grassman et al., 2023 ^38^. Gene set enrichment analyses were conducted using Partek Flow as above, (with a threshold of ± 2-fold change in expression and p-value < 0.05) to test for enrichment of dialysis membrane chamber or nymph adaptive genes.

#### PDI Functional Validations with Rickettsial Pathogens

##### Bacitracin treatment of host cells and TCEP rescue.

HeLa human cervical epithelial cells or Vero76 African green monkey kidney cells were seeded in 24-well plates with or without 12-mm round glass coverslips (Electron Microscopy Sciences, Hatfield, PA). Prior to infection, host cells were treated with 3 mM bacitracin (ThermoFisher; J62432.06) or vehicle (molecular grade H2O) for 1 h at 37°C in a humidified incubator with 5% atmospheric CO_2_. Host cell-free *O. tsutsugamushi* Ikeda or *R. montanensis* M5/6 organisms isolated as described previously ^102,111^ were treated with 0.01 mM tris(2-carboxyethyl)phosphine-HCl (TCEP, ThermoFisher; 20490) or vehicle (molecular grade H2O) for 30 minutes with rotation at room temperature followed by washing with PBS. After treatment, bacteria were resuspended in complete media and used to infect HeLa or Vero 76 cells as described previously ^102,111,112^. At 2 hpi, the cells were fixed in 4% (vol/vol) paraformaldehyde in PBS for 15 minutes. Extracellular bacteria were immunolabeled by incubation with either rabbit-anti *O. tsutsugamushi* TSA56 (56-kDa type-specific antigen; ^113^ antiserum at 1:1,000 or guinea pig anti-*R. rickettsii* (Sheila Smith) serum (that cross reacts with other spotted fever group rickettsiae, including *R. montanensis*) at 1:20 in PBS containing 5% (vol/vol) BSA for 60 minutes. The cells were washed 3 times with PBS. Secondary antibodies used were Alexa Fluor 488-conjugated chicken anti-rabbit IgG (Invitrogen A-21441; RRID:AB_2535859) or goat anti-guinea pig IgG (Invitrogen; A-11073; RRID:AB_2534117) at 1:1,000 in PBS containing 5% BSA. Next, to detect both extracellular and intracellular bacteria, the cells were permeabilized with 0.1% (vol/vol) triton X-100 in PBS for 10 minutes. After permeabilization, samples were immunolabeled with primary antibodies to detect *O. tsutsugamushi* or *R. montanensis* M5/6 as described above. Secondary antibodies used were Alexa Fluor 594-conjugated goat anti-rabbit IgG (Invitrogen; A-11012; RRID:AB_2534079) or goat anti-guinea pig IgG (Invitrogen; A-11076; RRID:AB_2534120) at 1:1,000 with the addition of 0.1 mg/ml 4′,6′-diamidino-2-phenylindole (Invitrogen; D1306) in PBS containing 5% (vol/vol) BSA for 60 minutes followed by PBS washing. Coverslips were mounted using ProLong Gold antifade reagent (Invitrogen; P36930). The cells were imaged at room temperature with a TCS SP8 microscope (Leica Microsystems, Germany). The number of intracellular bacteria per host cell was quantified by manually counting bacteria that were Alexa Fluor 594-positive but Alexa Fluor 488-negative in merged immunofluorescence micrographs visualized using Fiji software (https://fiji.sc/). Representative confocal images were acquired using a Zeiss LSM 700 laser scanning confocal microscope (Zeiss, Germany). To determine the effects of bacitracin treatment and TCEP rescue on *O. tsutsugamushi* growth at later timepoints of infection, bacitracin treatment of HeLa cells and TCEP treatment of *O. tsutsugamushi* was performed as described ^45^. Lysates were collected at 48 and 72 hpi by boiling samples in H2O at 95°C for 15 minutes ^114^. qPCR was performed to quantify *O. tsutsugamushi* genomic equivalents using oligonucleotides targeting *tsa56* (*tsa56*-F [5’-gttactgcattgtcacatgctaat-3’] and *tsa56*-R [5’-gaacttcaatattagctaccttagcga-3’]) and PerfeCTa SYBR Green Fastmix (Quantbio; 95072–250). Thermal cycling conditions were 95°C for 30 seconds followed by 40 cycles of 95°C for 10 seconds to 54°C for 10, and a 65°C to 95°C melt curve.

##### Analysis of bacitracin antibacterial effects.

Host cell-free *O. tsutsugamushi* or *R. montanensis* M5/6 were treated with 3 mM bacitracin or vehicle for 1 hour at 37°C followed by incubation with untreated HeLa or Vero 76 cells, respectively. Total DNA collected at 24, 48, and 72 hpi was subjected to qPCR to measure genomic equivalents of *O. tsutsugamushi* as described above or *R. montanensis* M5/6 using primers targeting the *sca1* gene ^115^.

### QUANTIFICATION AND STATISTICAL ANALYSIS

#### Identification of pathogen-recognizing proteins.

Our previous screens used a BASEHIT score calculation that was developed for commensal microbes ^18^ and intended to have high stringency, given the lower number of expected interactions between commensals and host extracellular proteins ^16,17^. With pathogens, however, we found this method incorrectly penalized scores for many previously characterized and potentially novel hits because the algorithm led to the interpretation of the higher reactivity of pathogens and human proteins as “non-specific” binding. Thus, we developed an alternative BASEHIT scoring calculation for pathogens that accounts for the higher number of expected interactions (Fig. S1A). The protein-producing clone-specific barcodes in the sequencing output were counted for each sample and the pre-selection library. These barcode counts were then subjected to linear normalization factors based on the average reads per sample in a sequencing run, barcode counts in plate-specific streptavidin coated magnetic bead-alone negative controls, screen-specific pre-selection library negative controls, and the average enrichment of each protein-producing clone (Fig. S1).

#### Hierarchical clustering of samples based on interaction profiles.

The interaction profiles of each pathogen sample were analyzed by the R package ade4 ^116^ to determine the Jaccard similarity index. The distance (1 - coefficient) was then determined for each sample, and samples were clustered using the complete linkage method. The interactions with proteins enriched by two or more pathogen samples were also visualized using Cytoscape ^117^.

#### Gene Ontology enrichment analyses.

Gene Ontology (GO) biological pathway (BP), molecular function (MF), and cellular component (CC) enrichment analyses were conducted on the DAVID Functional Classification Tool server ^118,119^ by mapping to Uniprot accessions with the list of sample-specific hits input as the analyzed gene list and the overall BASEHIT library input as the background. Significant enrichment was determined by an EASE p-value of < 0.05. Analyses of significantly enriched GOTerms were conducted using the DAVID GOTerm categories of BP, CC, and MF_Direct. Clusters of significantly enriched GOTerms were identified using the DAVID GOTerm categories of BP and MF_FAT. We clustered any redundant terms by a kappa statistic to group together terms that shared enriched proteins (K > 0.5).

#### Other statistical analyses.

Unless otherwise noted, other comparisons were performed using Prism 9.0 (GraphPad, San Diego, CA). One-way analysis of variance followed by Tukey’s post hoc was used to test for significant differences between groups. Statistical significance was set at p-values of < 0.05.

## Supplementary Material

1 **Table S1**. Human extracellular and secreted proteins included in the BASEHIT library, Related to STAR Methods.

2 **Table S2**. Proteins identified as putative binding partners for each pathogen sample, Related to Figures 1–4.

3 **Table S3**. Differentially regulated *B. burgdorferi* genes by EGF treatment, Related to Figure 3.

4 **Figure S1. Pathogen BASEHIT analysis algorithm**, Related to STAR Methods. **(A)** The protocol for pathogen BASEHIT computational enrichment algorithm is shown and detailed. The protein-specific barcode counts (“bcs”) for each screen is normalized to the number of reads/sample, the equivalent barcode counts in the preselection and bead-alone negative control samples, and the average enrichment of the protein. Any proteins with a normalized enrichment score above a pathogen-specific threshold is then called as a hit.

5 **Figure S2. Identification of pathogen-specific enrichment score thresholds**, Related to STAR Methods **(A-L)** Normalized enrichment thresholds were determined for each pathogen (gray dotted line). The total number of hits (orange), the number of known binding partners (purple) were determined for thresholds ranging from 2–10.

6 **Figure S3. Further analysis of BASEHIT screens**, Related to Figure 1. **(A)** A dendrogram illustrating the Jaccard distance based on interacting proteins for each sample demonstrates pathogens of the same taxonomic groupings had similar interaction profiles. Samples are annotated as [species]_[strain]_[treatment], and are colored by genus. *Orientia tsutsugamushi* (“Ot”) were isolated extracellularly (“E”) and intracellularly (“I”). Lyme *Borrelia* were grown at 33°C then shifted for 24 hours to 23°C (tick temperature), 37°C (host temperature), or 26°C, 30°C, or 33°C (intermediary temperatures). *Leptospira interrogans* (“Li), *L. licerasiae* (“Ll”), and *L. biflexa* (“Lb”) were grown at 30°C, then shifted for 4 hours to 37°C + 120 mM NaCl (“NaCl”; simulating host) or 30°C (standard culture condition). *Plasmodium falciparum* (“Pf”) and *P. berghei* (“Pb”) sporozoites (“sporo”) were isolated from the midguts (“MG”) or salivary glands (“SG”) of *Anopheles gambiae* (“Ag”) or *A. stephensii* (“As”). *P. falciparum* trophozoites (“troph”) and gametocytes (“gamet”) were cultured *in vitro*. Gametocytes were cultured after 9 (“d9”) and 15 (“d15”) days of growth. **(B)** GOTerms significantly enriched among hits with all the pathogen samples that failed to be grouped into larger clusters are shown plotted against -Log_10_(p-value) (DAVID EASE p-value < 0.05, K > 0.5). The legend below shows the GOTerms plotted. **(C)** The binding of SLPI to *Borrelia burgdorferi* N40 was validated by ELISA. Lysate of 10^7^ spirochetes were coated on plates, followed by 1 (orange) or 100 (green) ng of human IgG (hIgG) or Fc-conjugated SLPI. Binding was determined by HRP-conjugated anti-human Fc antibody. Significance (*) was determined by two-tailed Mann-Whitney test. **(D)** Flow cytometric validations corroborated binding of *Borrelia burgdorferi* N40 to IL28a, IL29, and BTLA, but not LAIR1. 10^7^ *Borrelia burgdorferi* N40 were incubated with 2 ug of recombinant His-tagged protein or no protein (negative control; “Ab only”), followed by an anti-6XHis monoclonal antibody conjugated to Alexa Fluor 488. **(E)** A heat map illustrating the p-values of GO BPs (y-axis) significantly enriched among human proteins interacting with *A. phagocytophilum*, *B. duncani*, *R. rickettsia*, *R. typhi*, and *R. montanensis, O. tsutsugamushi, C. burnetti*, and *P. falciparum* and *P. berghei* (x-axis) is shown. *P. falciparum* and *P. berghei* are split into gametocytes and trophozoites (“blood stages”) and sporozoites. P-values ≤ 0.05 are shown in blue, and p-values > 0.05 are shown in a gradient from white to black. lotted against -Log_10_(p-value). Significance was determined by DAVID with EASE (p-value < 0.05).

7 **Figure S4. Functional analysis of select hits**, Related to Figures 3 and 5. **(A)** Growth curves demonstrating EGF does not influence *Borrelia* viability at 33 or 37°C. *B. burgdorferi* was grown at 33°C (blue) or 37°C (red) for 7 days. 10nM of Fc-tagged EGF (circle) or the Fc tag alone (triangle) was added every 24 hours, and *Borrelia* were counted. Shown at each timepoint are the mean counts of 3 cultures ± the standard error of the mean. No differences were found in the exponential growth curves of Fc and EGF at 33°C or at 37°C (Sum-of-squares F-test, p-values = 0.28 and 0.49, respectively). **(B)** Gene set enrichment analysis comparing genes up or downregulated by EGF treatment at 33 and 37°C uncovered that EGF treatment significantly upregulated *B. burgdorferi* genes expressed by spirochetes in dialysis membrane chambers (Pink, “DMC”) and downregulated genes expressed by spirochetes in feeding nymphs (Yellow, “Nymph”) ^38^. Closed circles indicate significant enrichment (p-value < 0.05)**. (C)** *R. montanensis* and **(D)** *O. tsutsugamushi* were treated with 3mM bacitracin (“Bac”) or Water (“Veh”) for 1 hour, washed, then seeded on HeLa or Vero76 cells, respectively. Bacterial genomes per ml culture were then determined by qPCR.

## Figures and Tables

**Table 1.** Pathogens and their growth treatments used to screen the BASEHIT library.

Pathogen species	Strain/serovar	Treatment/life stage
*Borrelia burgdorferi*	N40, B31	23, 26, 30, 33, 37°C
	HP19, CA8, CT-1, VS134, NT-1	33°C
*B. afzelii*	CB43	23, 26, 30, 33, 37°C
	TV38, TV34	33°C
*B. garinii*	G25	23, 26, 30, 33, 37°C
	TV39	33°C
*B. bavariensis*	PBi, PNi	33°C
*Leptospira interrogans*	Manilae L495, Copenhageni L1–130, Canicola, Lai 56601	30°C and 0 mM or 37°C and 100 mM NaCl
*L*. *licerasiae*	Varillal VAR010	30°C and 0 mM or 37°C and 100 mM NaCl
*L. biflexa*	Patoc	30°C and 0 mM or 37°C and 100 mM NaCl
*Plasmodium falciparum*	NF54	Trophozoites grown *in vitro*
	NF54	Gametocytes isolated after 9 or 15 days of *in vitro* growth
	NF54	Sporozoites from *Anopheles gambiae* midguts with and without density gradient
	NF54	Sporozoites isolated from *Anopheles gambiae* and *A. stephensi* salivary glands with and without density gradient
*P. berghei*	ANKA	Sporozoites isolated from *Anopheles gambiae* midguts by fraction
	ANKA	Sporozoites isolated from *Anopheles gambiae* salivary glands
*Orientia tsutsugamushi*	TA768, Gilliam, TA686, Ikeda, UT76, Karp (two isolates), Kato	Intracellular and extracellular isolates from L929 cells
	Karp	Intracellular and extracellular isolates from HeLa cells
	Ikeda	Intracellular isolates from HeLa cells
*Anaplasma phagocytophilum*	NCH1, HZ	
*Babesia duncani*	WAI	
*Chlamydia trachomatis*	L2	
*Coxiella burnetii*	NMII	
*Ehrlichia chaffeensis*	Arkansas	
*Francisella tularensis*	LVS	
*Rickettsia montanensis*	M5/6	
*R. rickettsii*	Sheila Smith	
*R. typhi*	Wilmington	

**Table:** KEY RESOURCES TABLE

REAGENT or RESOURCE	SOURCE	IDENTIFIER
Antibodies		
PE anti-DYKDDDDK Tag Antibody	BioLegend	Cat no.: 637309; RRID:AB_2563147
HRP Anti-6X His tag antibody	abcam	Cat. no.: ab1187; RRID:AB_298652
Alexa Fluor 488 Anti-6x His Tag Monoclonal Antibody	ThermoFisher	Cat. no.: MA1-21315-A488; RRID:AB_2610645
AVP Polyclonal Antibody	ThermoFisher	Cat. no.: PA5-112845; RRID:AB_2867579
F(ab')2-Donkey anti-Rabbit IgG (H+L) Secondary Antibody, PE, eBioscience	ThermoFisher	Cat. no.: 12-4739-81; RRID:AB_1210761
Chicken anti-Rabbit IgG (H+L) Cross-Adsorbed Secondary Antibody, Alexa Fluor 488	ThermoFisher	Cat. no.: A-21441; RRID:AB_2535859
Goat anti-Guinea Pig IgG (H+L) Highly Cross-Adsorbed Secondary Antibody, Alexa Fluor^™^ 488	ThermoFisher	Cat. no.: A-11073; RRID:AB_2534117
Goat anti-Rabbit IgG (H+L) Cross-Adsorbed Secondary Antibody, Alexa Fluor^™^ 594	ThermoFisher	Cat. no.: A-11012; RRID:AB_2534079
Goat anti-Guinea Pig IgG (H+L) Highly Cross-Adsorbed Secondary Antibody, Alexa Fluor^™^ 594	ThermoFisher	Cat. no.: A-11076; RRID:AB_2534120
Rabbit-anti *O. tsutsugamushi* TSA56	Lab of Jason Carlyon; Beyer et al. ^113^	N/A
Bacteria and parasite strains		
*Anaplasma phagocytophilum* NCH1	Laboratory of Joao H.F. Pedra	NCBI:txid135916 1
*A. phagocytophilum* HZ	Laboratory of Jason A. Carlyon	NCBI:txid212042
*Borrelia burgdorferi* N40	Laboratory of Erol Fikrig	NCBI:txid521007
*B. burgdorferi* B31	Laboratory of Erol Fikrig	NCBI:txid224326
*B. burgdorferi* HP19	Laboratory of Melissa J. Caimano and Justin D. Radolf; Steere et al. ^87^	N/A
*B. burgdorferi* CA8	Laboratory of Melissa J. Caimano and Justin D. Radolf	NCBI:txid747491
*B. burgdorferi* CT-1	Laboratory of Melissa J. Caimano and Justin D. Radolf; Hughes et al. ^88^	N/A
*B. burgdorferi* VS134	Laboratory of Melissa J. Caimano and Justin D. Radolf; Peter et al. ^89^	N/A
*B. burgdorferi* NT-1	Laboratory of Melissa J. Caimano and Justin D. Radolf; Foley et al. ^90^	N/A
*B. afzelii* CB43	Laboratory of Erol Fikrig; Stepanova-Tresova et al. ^91^	N/A
*B. afzelii* TV34	Laboratory of Utpal Pal	N/A
*B. afzelii* TV38	Laboratory of Utpal Pal	N/A
*B. garinii* G25	Laboratory of Melissa J. Caimano and Justin D. Radolf; Godfroid et al. ^92^	N/A
*B. garinii* TV39	Laboratory of Utpal Pal	N/A
*B. bavariensis* PBi	Laboratory of Gabriele Margos	NCBI:txid290434
*B. bavariensis* PNi	Laboratory of Gabriele Margos; Rollins et al. ^93^	N/A
*Leptospira interrogans* Manilae L495	Laboratory of Joseph M. Vinetz	NCBI:txid214675
*L. interrogans* Copenhageni L1-130	Laboratory of Joseph M. Vinetz	NCBI:txid267671
*L. interrogans* Canicola	Laboratory of Joseph M. Vinetz	NCBI:txid211880
*L. interrogans* Lai 56601	Laboratory of Joseph M. Vinetz	NCBI:txid189518
*L*. *licerasiae* Varillal VAR010	Laboratory of Joseph M. Vinetz	NCBI:txid1049972
*L. biflexa* Patoc	Laboratory of Joseph M. Vinetz	NCBI:txid145259
*Plasmodium falciparum* NF54	Laboratories of George Dimopoulos and Abhai K. Tripathi	NCBI:txid5843
*P. berghei* ANKA GFPCON 259cl2	BEI Resources	Cat. no.: MRA-865; NCBI:txid5823
*Orientia tsutsugamushi* TA763	Laboratory of Jeanne Salje	NCBI:txid1359176
*O. tsutsugamushi* Gilliam	Laboratory of Jeanne Salje	NCBI:txid1359184
*O. tsutsugamushi* TA686	Laboratory of Jeanne Salje	NCBI:txid1359176
*O. tsutsugamushi* Ikeda	Laboratory of Jeanne Salje	NCBI:txid334380
*O. tsutsugamushi* UT76	Laboratory of Jeanne Salje	NCBI:txid682184
*O. tsutsugamushi* Karp	Laboratories of Jeanne Salje and Jason A. Carlyon	NCBI:txid1359185
*O. tsutsugamushi* Kato	Laboratory of Jeanne Salje	NCBI:txid1359186
*Babesia duncani* WAI	Laboratory of Choukri Ben Mamoun	NCBI:txid323732
*Chlamydia trachomatis* L2	ATCC	Cat. no.: VR-902B; NCBI:txid471472
*Coxiella burnetii* NMII	Laboratory of Craig Roy	NCBI:txid777
*Ehrlichia chaffeensis* Arkansas	Laboratory of Joao H.F. Pedra	NCBI:txid205920
*Francisella tularensis* LVS	Laboratory of Joao H.F. Pedra	NCBI:txid376619
*Rickettsia montanensis* M5/6	Laboratory of Abdu F. Azad	NCBI:txid1359200
*R. rickettsii* Sheila Smith	Laboratory of Abdu F. Azad	NCBI:txid392021
*R. typhi* Wilmington	Laboratory of Abdu F. Azad	NCBI:txid257363
Chemicals, peptides, and recombinant proteins		
tris(2-carboxyethyl)phosphine-HCl (TCEP)	ThermoFisher	Cat. no.: 20490
NEST1	Laboratory of Erol Fikrig; Marin-López et al. ^110^	N/A
Recombinant Human IL-28A/IFN-lambda 2 Protein	R&D Systems	Cat. no.: 1587-IL
Recombinant Human IL-29/IFN-lambda 1 Protein	R&D Systems	Cat. no.: 1588-IL
Recombinant Human BTLA His-tag Protein, CF	R&D Systems	Cat. no.: 9235-BT
Recombinant Human ULBP-2 His-tag Protein, CF	R&D Systems	Cat. no.: 11196-UL
Recombinant Human LAIR1 His Tagged Protein, CF	R&D Systems	Cat. no.: 2664-LR
[Arg8]-Vasopressin	R&D Systems	Cat. no.: 2935
HRP-Conjugated Streptavidin	ThermoFisher	Cat. no.: N100
EGF Fc Chimera, Human	GenScript	Cat. no.: Z03377
Accudenz	Accurate Chemical & Scientific Corporation	Cat. no.: AN7050
Sulfo-NHS-LC-Biotin	ThermoFisher	Cat. no: A39257
Experimental models: Cell lines		
BASEHIT library	Laboratory of Aaron M. Ring; Sonnert et al. ^18^	N/A
HL-60	ATCC	Cat. no.: CCL-240; RRID:CVCL_0002
Vero 76	ATCC	Cat. no.: CRL-1587; RRID:CVCL_0603
NCTC clone 929 [L cell, L-929, derivative of Strain L]	ATCC	Cat. no.: CCL-1; RRID:CVCL_0462
HeLa	ATCC	Cat. no.: CCL-2; RRID:CVCL_0030
DH82	ATCC	Cat. no.: CRL-3590; RRID:CVCL_2018
Experimental models: Organisms/strains		
*Anopheles gambiae* 4ARR	BEI Resources	Cat. no.: MRA-121; NCBI:txid7165
*A. gambiae* Keele	Laboratories of George Dimopoulos and Abhai K. Tripathi	NCBI:txid7165
A. stephensi Liston	Laboratories of George Dimopoulos and Abhai K. Tripathi	NCBI:txid30069
Oligonucleotides		
*O. tsutsugamushi tsa56:* Forward: gttactgcattgtcacatgctaat; Reverse: gaacttcaatattagctaccttagcga	This paper	N/A
*R. montanensis sca1:* Forward: CAAGCTCGTTATTACCCCGAAT; Reverse: CTACCGCTCCTTGGAATGTTAGACC	Curto et al. ^115^	N/A
Nextera i5 and i7 primers	Illumina	Cat. no.: FC-131-1096
Software and algorithms		
FlowJo	FlowJo	N/A
Partek Flow 10.0.23.0720	Partek	N/A
Prism 9.0	GraphPad	N/A
ade4	Dray et al. ^116^	N/A
DAVID	Sherman et al. ^118,119^	N/A
Cytoscape	Shannon et al. ^117^	N/A
Other		
Barbour-Stoenner-Kelly H (BSK-H) complete medium	Millipore Sigma	B8291-100ML
BD Difco Dehydrated Culture Media: Leptospira Medium Base EMJH	Fisher Scientific	Cat. no.: DF0794-17-1; BD Ref: 279410
Corning^®^ 500 mL RPMI 1640	Corning	Cat. no.: 10-041-CV
Dulbecco’s Modified Eagle Medium	ThermoFisher	Cat. no.: 21013
Streptavidin-coated magnetic beads	SpheroTech	Cat. no.: SVM-025-5H

## Data Availability

The BASEHIT analyses are available in this article and supplemental materials and code has been deposited in GitHub ^18^. Pathogen strains may be obtained through correspondence with Dr. Erol Fikrig.

## HIGHLIGHTS

- A curated yeast display library of human proteins was probed with diverse pathogens
- 1,303 interactions were identified, and several were validated orthogonally
- Epidermal growth factor induced stage-specific gene expression in Lyme *Borrelia*
- Protein disulfide isomerases facilitated cell invasion by Rickettsial pathogens
