# Supplementary material for: An atlas of human vector-borne microbe interactions reveals pathogenicity mechanisms
Source: Cell. Author manuscript; Available in PMC 2025 Jul 25. (PMC11959484; doi:10.1016/j.cell.2024.05.023)
Supplement: 4 — Figure S1. Pathogen BASEHIT analysis algorithm, Related to STAR Methods. (A) The protocol for pathogen BASEHIT computational enrichment algorithm is shown and detailed. The protein-specific barcode counts (“bcs”) for each screen is normalized to the number of reads/sample, the equivalent barcode counts in the preselection and bead-alone negative control samples, and the average enrichment of the protein. Any proteins with a normalized enrichment score above a pathogen-specific threshold is then called as a hit. [file NIHMS1996746-supplement-4.pdf]

Fig. S1

A

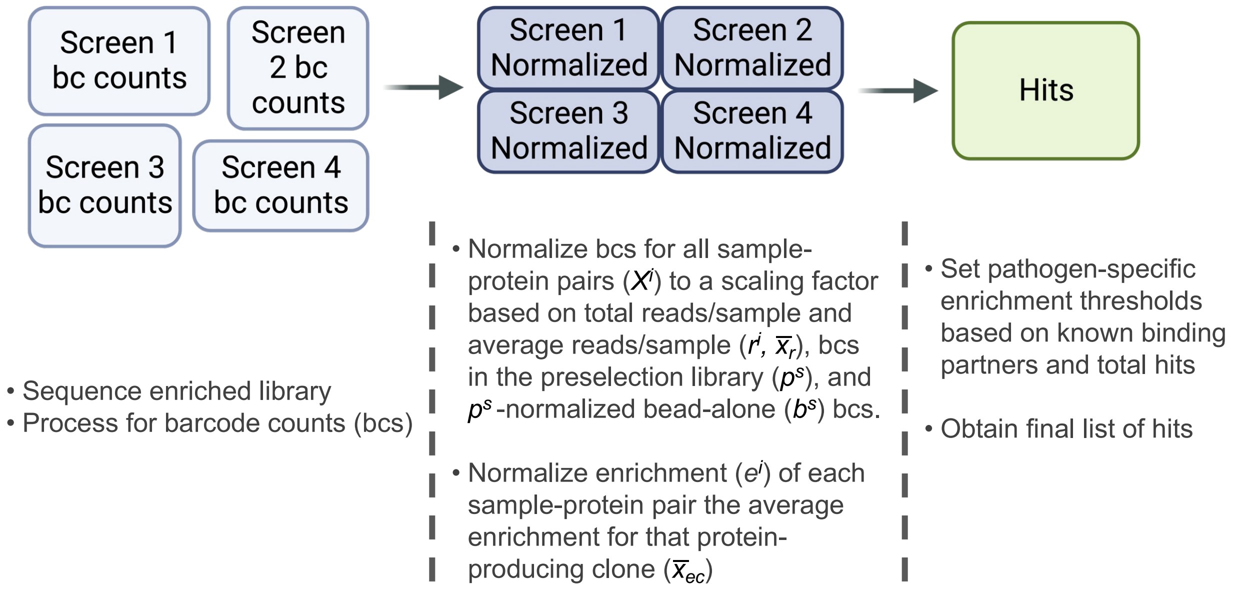

$$Normalized\ barcode\ count = X^s = \left(\frac{X^i}{r^i/\overline{x}_r}\right)$$

$$Enrichment = e = \left(\frac{X^s/b^s}{p^s}\right)$$

$$Normalized\ Enrichment = \left(e^i/\overline{x}_{ec}\right)$$
