# Supplementary figures and images for: An atlas of human vector-borne microbe interactions reveals pathogenicity mechanisms
Source: Cell. Author manuscript; Available in PMC 2025 Jul 25. (PMC11959484; doi:10.1016/j.cell.2024.05.023)

Fig. S2

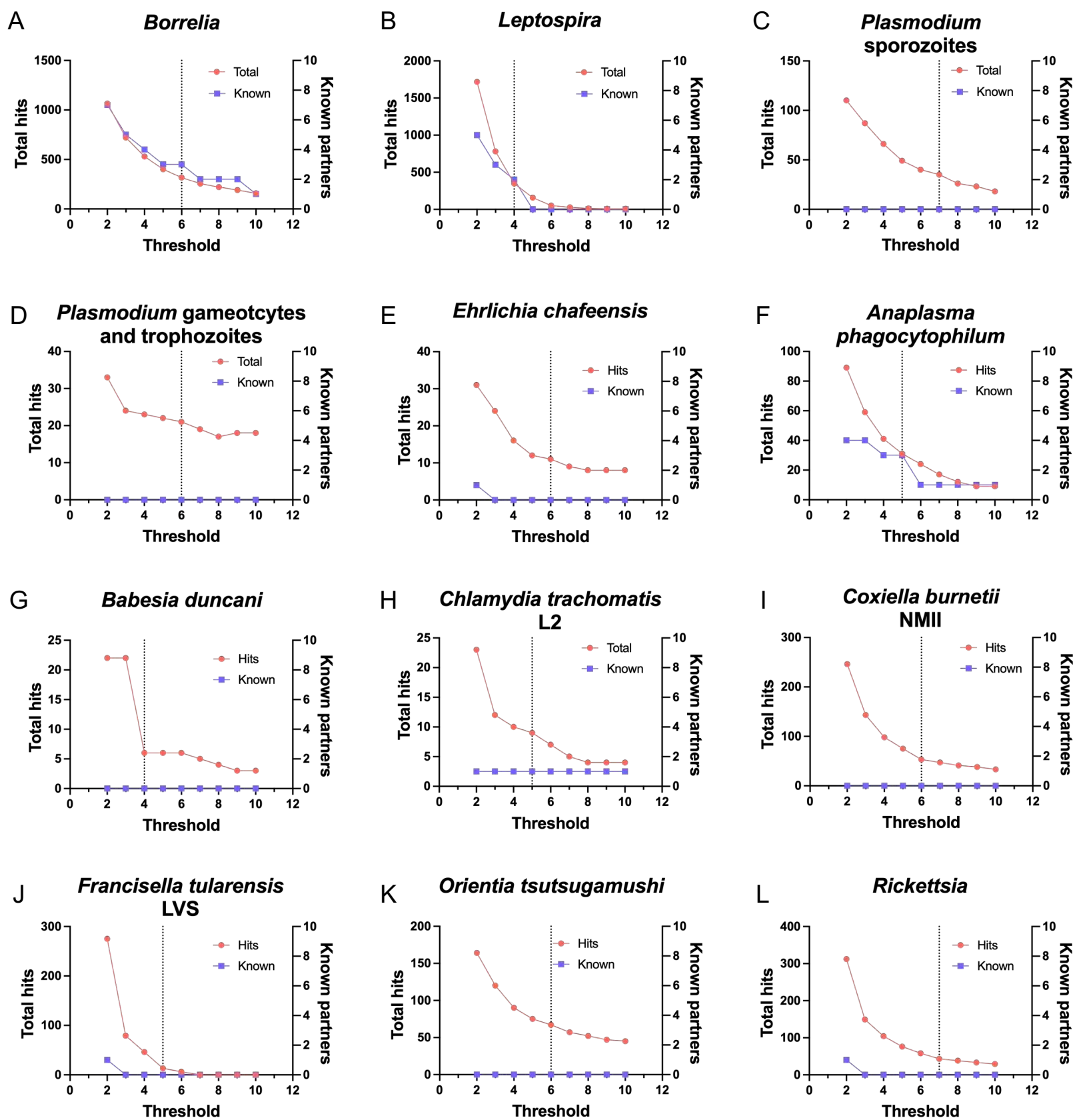

Supplement: 5 — Figure S2. Identification of pathogen-specific enrichment score thresholds, Related to STAR Methods (A-L) Normalized enrichment thresholds were determined for each pathogen (gray dotted line). The total number of hits (orange), the number of known binding partners (purple) were determined for thresholds ranging from 2–10. [file NIHMS1996746-supplement-5.pdf]

Fig. S3

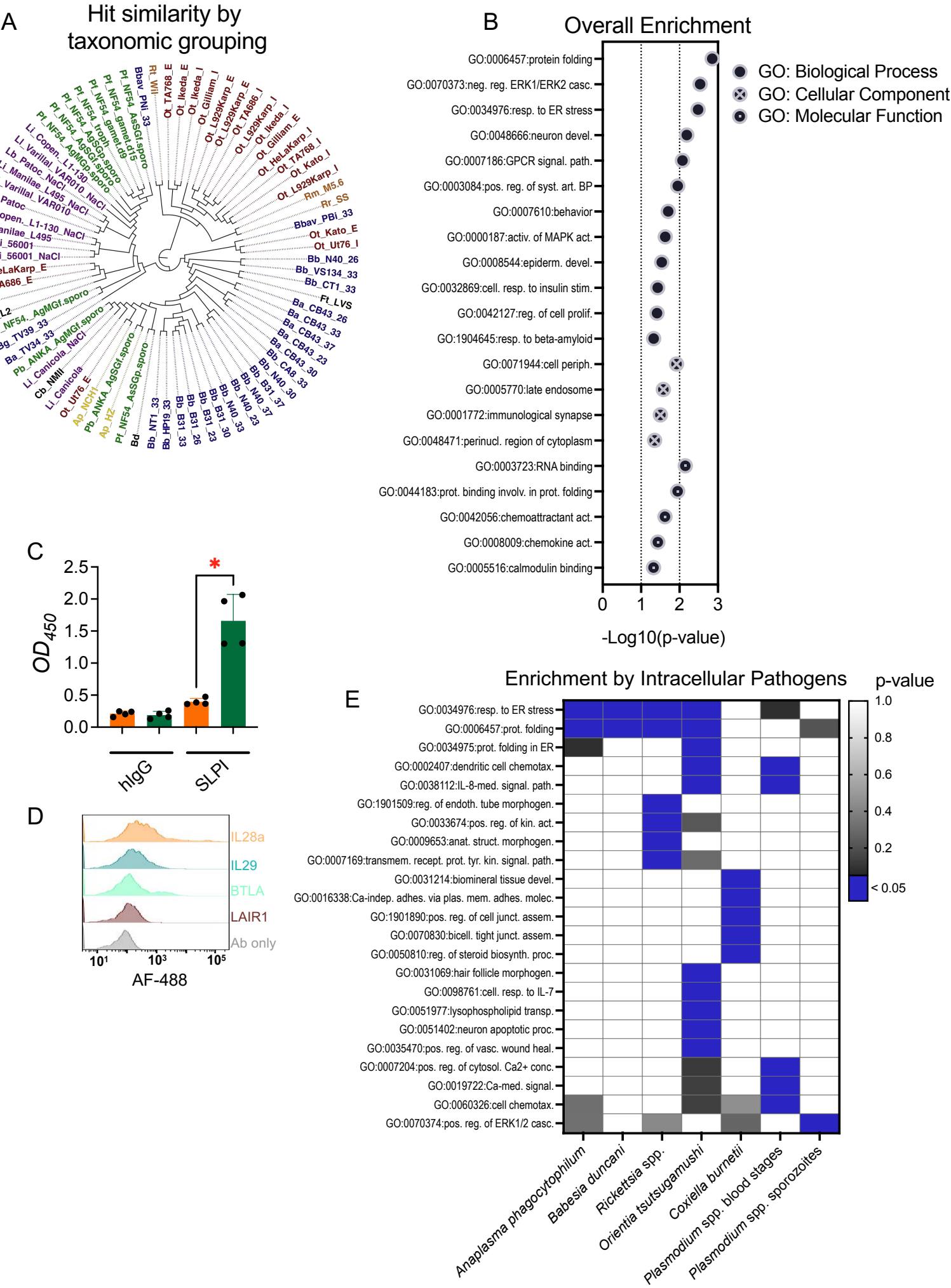

Supplement: 6 — Figure S3. Further analysis of BASEHIT screens, Related to Figure 1. (A) A dendrogram illustrating the Jaccard distance based on interacting proteins for each sample demonstrates pathogens of the same taxonomic groupings had similar interaction profiles. Samples are annotated as [species]_[strain]_[treatment], and are colored by genus. Orientia tsutsugamushi (“Ot”) were isolated extracellularly (“E”) and intracellularly (“I”). Lyme Borrelia were grown at 33°C then shifted for 24 hours to 23°C (tick temperature), 37°C (host temperature), or 26°C, 30°C, or 33°C (intermediary temperatures). Leptospira interrogans (“Li), L. licerasiae (“Ll”), and L. biflexa (“Lb”) were grown at 30°C, then shifted for 4 hours to 37°C + 120 mM NaCl (“NaCl”; simulating host) or 30°C (standard culture condition). Plasmodium falciparum (“Pf”) and P. berghei (“Pb”) sporozoites (“sporo”) were isolated from the midguts (“MG”) or salivary glands (“SG”) of Anopheles gambiae (“Ag”) or A. stephensii (“As”). P. falciparum trophozoites (“troph”) and gametocytes (“gamet”) were cultured in vitro. Gametocytes were cultured after 9 (“d9”) and 15 (“d15”) days of growth. (B) GOTerms significantly enriched among hits with all the pathogen samples that failed to be grouped into larger clusters are shown plotted against -Log10(p-value) (DAVID EASE p-value < 0.05, K > 0.5). The legend below shows the GOTerms plotted. (C) The binding of SLPI to Borrelia burgdorferi N40 was validated by ELISA. Lysate of 107 spirochetes were coated on plates, followed by 1 (orange) or 100 (green) ng of human IgG (hIgG) or Fc-conjugated SLPI. Binding was determined by HRP-conjugated anti-human Fc antibody. Significance (*) was determined by two-tailed Mann-Whitney test. (D) Flow cytometric validations corroborated binding of Borrelia burgdorferi N40 to IL28a, IL29, and BTLA, but not LAIR1. 107 Borrelia burgdorferi N40 were incubated with 2 ug of recombinant His-tagged protein or no protein (negative control; “Ab only”), followed b [file NIHMS1996746-supplement-6.pdf]

Fig. S4

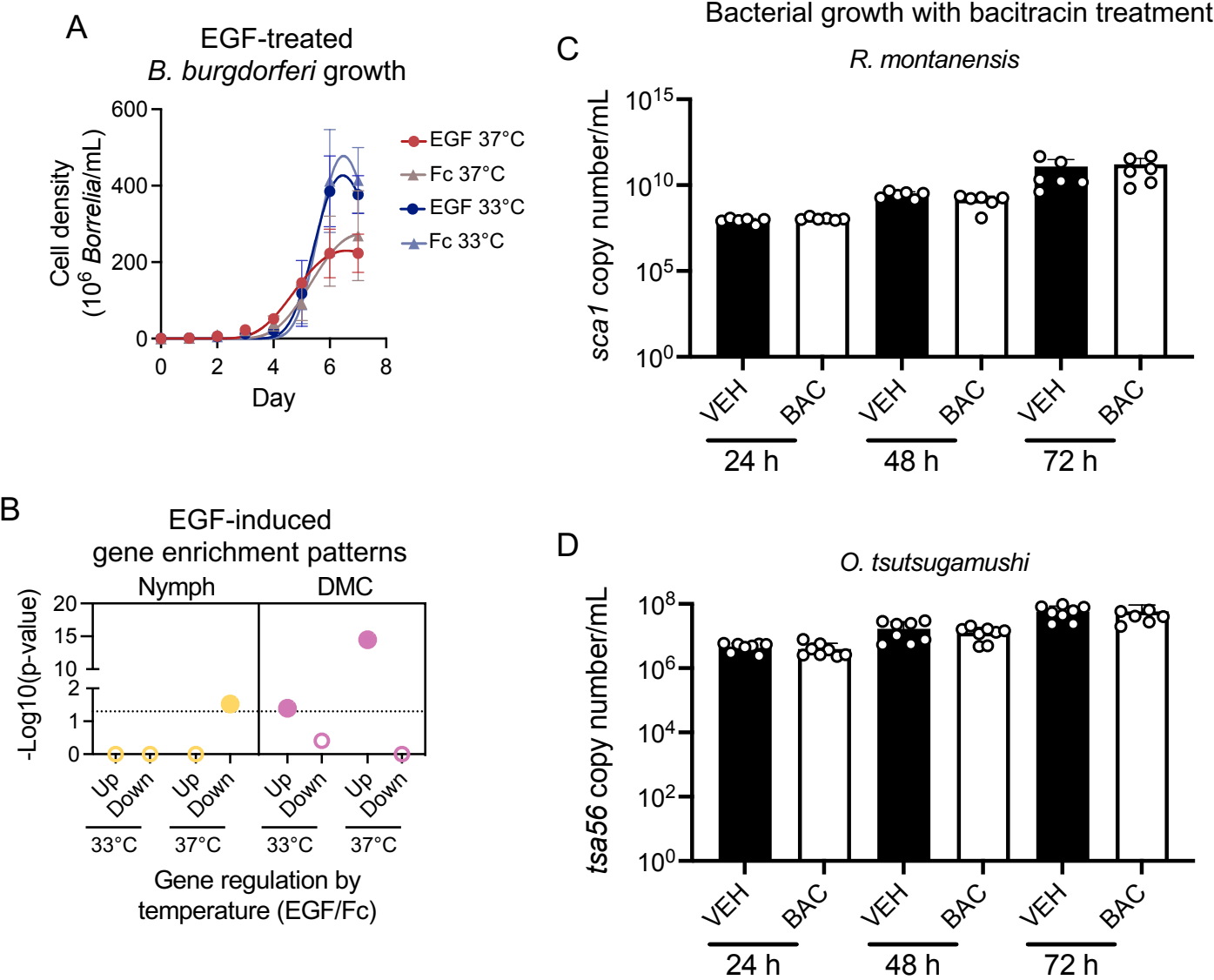

Supplement: 7 — Figure S4. Functional analysis of select hits, Related to Figures 3 and 5. (A) Growth curves demonstrating EGF does not influence Borrelia viability at 33 or 37°C. B. burgdorferi was grown at 33°C (blue) or 37°C (red) for 7 days. 10nM of Fc-tagged EGF (circle) or the Fc tag alone (triangle) was added every 24 hours, and Borrelia were counted. Shown at each timepoint are the mean counts of 3 cultures ± the standard error of the mean. No differences were found in the exponential growth curves of Fc and EGF at 33°C or at 37°C (Sum-of-squares F-test, p-values = 0.28 and 0.49, respectively). (B) Gene set enrichment analysis comparing genes up or downregulated by EGF treatment at 33 and 37°C uncovered that EGF treatment significantly upregulated B. burgdorferi genes expressed by spirochetes in dialysis membrane chambers (Pink, “DMC”) and downregulated genes expressed by spirochetes in feeding nymphs (Yellow, “Nymph”) 38. Closed circles indicate significant enrichment (p-value < 0.05). (C) R. montanensis and (D) O. tsutsugamushi were treated with 3mM bacitracin (“Bac”) or Water (“Veh”) for 1 hour, washed, then seeded on HeLa or Vero76 cells, respectively. Bacterial genomes per ml culture were then determined by qPCR. [file NIHMS1996746-supplement-7.pdf]
